# An introgressed gene causes meiotic drive in *Neurospora sitophila*

**DOI:** 10.1073/pnas.2026605118

**Published:** 2021-04-19

**Authors:** Jesper Svedberg, Aaron A. Vogan, Nicholas A. Rhoades, Dilini Sarmarajeewa, David J. Jacobson, Martin Lascoux, Thomas M. Hammond, Hanna Johannesson

**Affiliations:** ^a^Department of Organismal Biology, Evolutionary Biology Centre (EBC), Uppsala University, 752 36 Uppsala, Sweden;; ^b^Department of Biomolecular Engineering, University of California, Santa Cruz, CA 95064;; ^c^School of Biological Sciences, Illinois State University, Normal, IL 61790;; ^d^Department of Ecology and Genetics, EBC, Uppsala University, 752 36 Uppsala, Sweden

**Keywords:** meiotic drive, genomic conflict, spore killer, *Neurospora*

## Abstract

Meiotic drive elements cause their own preferential transmission following meiosis. In fungi, this phenomenon takes the shape of spore killing, and in the filamentous ascomycete *Neurospora sitophila*, the *Sk-1* spore killer element is found in many natural populations. In this study, we identify the gene responsible for spore killing in *Sk-1* by generating both long- and short-read genomic data and by using these data to perform a genome-wide association test. We name this gene *Spk-1*. Through molecular dissection, we show that a single 405-nt-long open reading frame generates a product that both acts as a poison capable of killing sibling spores and as an antidote that rescues spores that produce it. By phylogenetic analysis, we demonstrate that the gene has likely been introgressed from the closely related species *Neurospora hispaniola*, and we identify three subclades of *N. sitophila*, one where *Sk-1* is fixed, another where *Sk-1* is absent, and a third where both killer and sensitive strain are found. Finally, we show that spore killing can be suppressed through an RNA interference-based genome defense pathway known as meiotic silencing by unpaired DNA. *Spk-1* is not related to other known meiotic drive genes, and similar sequences are only found within *Neurospora*. These results shed light on the diversity of genes capable of causing meiotic drive, their origin and evolution, and their interaction with the host genome.

## Significance

In order to survive, most organisms must deal with parasites. Such parasites can be other organisms or, sometimes, selfish genes found within the host genome itself. While much is known about parasitic organisms, the interaction with their hosts, and their ability to spread within and between species, much less is known about selfish genes. We here identify a selfish “spore killer” gene in the fungus *Neurospora sitophila*. The gene appears to have evolved within the genus but has entered the species through hybridization and introgression. We also show that the host can counteract the gene through RNA interference. These results shed light on the diversity of selfish genes in terms of origin, evolution, and host interactions.

Some genes can spread in a population even though they have negative effects on the organisms that carry them. These are often referred to as “selfish” genes and it is becoming increasingly clear that such genes can be important drivers of a large number of evolutionary patterns (1–3). One class of selfish genetic elements are killer meiotic drivers (KMDs). When heterozygous at meiosis they can increase their own transmission by destroying or incapacitating meiotic products that do not carry them. KMDs have been found across eukaryotes, and in animals and plants they generally act in male meiosis by killing sperm. In fungi they kill sexual spores, and are therefore known as “spore killers” (4, 5).

A KMD must perform two tasks: kill noncarrier (sensitive) meiotic products, and ensure that it does not kill itself. It can do this through either a poison–antidote mechanism, where they produce a poison that kills indiscriminately and an antidote that they keep to themselves, or through a killer–target mechanism, where the killer attacks a target element that can only be found in sensitive meiotic products. In order to successfully drive, the poison and antidote must always be inherited together and a target must never be inherited with a killer. For this reason many KMDs are found in regions of low recombination, such as inversions or on sex chromosomes (6–8). Some KMDs only require a single gene to function, and so are not associated with regions of low recombination. This is often the case in fungi, where the majority of identified KMDs are single-gene systems. In *Podospora*, a family of KMD genes named *Spok* are found at high frequencies (9, 10). *Spok* genes produce a single protein that can act as both poison and antidote simultaneously, probably through the action of different functional domains (10). *Podospora anserina* also harbors *het-s*, a single-gene KMD which produces a prion that can cause drive by targeting the protein generated from the sensitive allele (11). In *Schizosaccharomyces pombe*, the large and highly diverse *wtf* gene family causes drive by producing poison and antidote products from overlapping transcripts generated from two different start codons (12, 13). Strains of *S. pombe* vary in *wtf* content and a single strain can carry up to 14 different driving *wtf* genes, generating extensive sexual incompatibilities between even closely related isolates (14, 15).

As exemplified by the *wtf* genes, meiotic drive can often reduce the fertility of the organism. This puts the KMD in conflict with the rest of the genome, and suppressors are therefore expected to evolve. Suppression of drive has been observed in a number of species, but only in a few cases has the underlying mechanism of suppression been identified. For instance, in *Drosophila simulans*, RNA interference (RNAi) is necessary for suppression of a sex ratio KMD (16). The reduction of fertility is also hypothesized to drive reproductive isolation between populations, and some KMDs act as hybrid incompatibility loci (17–20). At the same time, if KMDs can cross reproductive barriers, they may be able to quickly establish themselves in sensitive populations and bring linked genes with them (21, 22).

In the ascomycete fungus *Neurospora*, three different spore killer KMDs have been identified (23). *Sk-2* and *Sk-3* are found in *Neurospora intermedia* and are both poison–antidote systems located in large, highly rearranged, nonrecombining regions (8, 24). The *rsk* gene performs the antidote function for both *Sk-2* and *Sk-3*, but the gene performing the poison function in *Sk-2* (*rfk-1*) is not found in *Sk-3* (8, 25, 26), meaning that an unknown gene must be responsible.

**Fig. 1.**
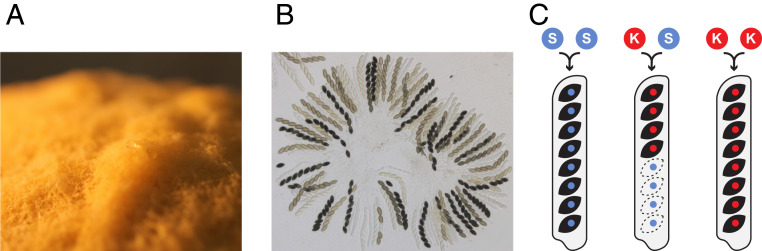
*N. sitophila* and *Sk-1* spore killing. (*A*) *N. sitophila* is closely related and morphologically similar to the model organism *N. crassa*. Both species produce large amounts of orange or yellow asexual spores (conidia). (*B*) Sexual ascospores in *N. sitophila*. *N. sitophila* is an obligate outcrosser and sexual reproduction takes place after a brief diploid stage when two haploid individuals encounter each other and cross. Each meiosis takes place in a cell known as an ascus and normally eight black, haploid ascospores are formed. (*C*) Schematic of produced ascospores in different crosses. In crosses which are homozygous for either the sensitive genotype (S-S) or the killer *Sk-1* genotype (K-K) eight black, viable ascospores are formed. In heterozygous crosses between sensitive and *Sk-1* killer strains (S-K), only four black, viable ascospores are formed. All surviving spores carry the *Sk-1* genotype and the sensitive spores degenerate during development and remain translucent. Spore killing occurs shortly after the cell walls surrounding the spores start to form. Homozygous *Sk-1* crosses produce eight spores as all spores carry a resistance factor which protects them from being killed.

*Sk-1* is found in *Neurospora sitophila* (Fig. 1 *A* and *B*) and was the first spore killer to be identified in *Neurospora*. In heterozygous *Sk-1* x sensitive crosses, nearly all spores carrying the sensitive genotype are killed, generating a distinct pattern of four black, viable spores and four white, dead spores (Fig. 1*C*) (23). Little is known about the genetics of *Sk-1* and its relationship to *Sk-2* and *Sk-3*, but an extensive sampling effort has identified and isolated over a hundred strains carrying *Sk-1*. While the *Sk-1* element is found globally and in ∼15% of natural *N. sitophila* isolates, the variation in frequency among locations is large. The element appears to be absent in many populations, fixed in others, and, in two well-sampled locations, Italy and Tahiti, both killers and sensitives are found in roughly equal frequencies (27, 28).

In this study, we set out to identify the locus responsible for spore killing in *N. sitophila*. Using whole-genome sequencing of 56 *N. sitophila* strains, we show that *Sk-1* spore killing is caused by a single gene (*Spk-1*) and demonstrate that it is responsible for both killing and resistance. Our data suggest that *Spk-1* has been introgressed from a different *Neurospora* species, potentially *Neurospora hispaniola*. Furthermore, we show that the population structure of *N. sitophila* is divided into three subclades, one of which is fixed for *Sk-1*, one where *Sk-1* is absent, and a third where killers and sensitives intermix. Finally, we show that spore killing can be suppressed in certain crosses by an RNAi-based genome defense mechanism known as meiotic silencing by unpaired DNA (MSUD). *Spk-1* is a class of spore killer genes and we demonstrate that RNAi can protect against meiotic drivers in fungi.

## Results

### The Locus Responsible for Spore Killing Is Located on Chromosome 6.

We sequenced four *N. sitophila* strains (two *Sk-1* killers and two sensitives) using the PacBio RSII platform, generating high-quality genome assemblies, two of which had most chromosomes assembled from end to end (*SI Appendix*, Table S1). Aligning these assemblies to the *Neurospora crassa* OR74 genome assembly (29) reveals that all four strains are largely collinear and that the two *Sk-1* strains do not carry any large rearrangements (*SI Appendix*, Fig. S1), unlike the *Sk-2* and *Sk-3* spore killer elements in *N. intermedia* (8).

We also generated short-read, paired-end sequencing data from 56 *N. sitophila* strains, using the Illumina HiSeq platform. Our sampling strategy aimed to capture the global diversity of *N. sitophila* and to include a balanced representation of both killer and sensitive strains (*SI Appendix*, Fig. S2 and Table S2). Special attention was paid to two well-sampled populations in Tahiti (28) and Italy (27), which were both polymorphic for *Sk-1*.

**Fig. 2.**
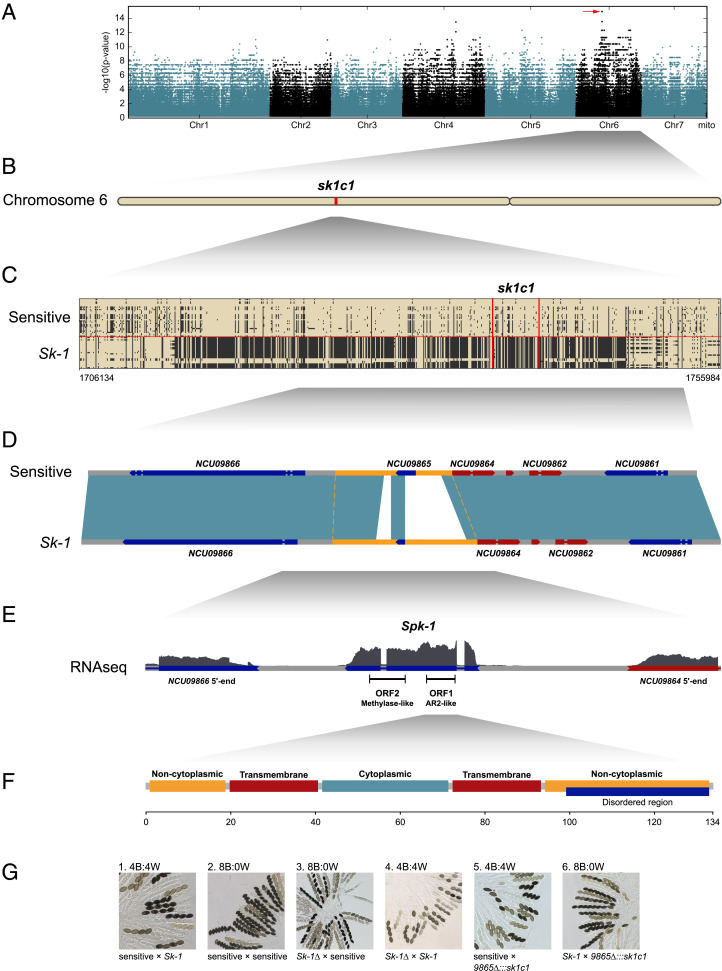
Locus responsible for spore killing. (*A*) Manhattan plot showing the association of 1,867,059 biallelic SNPs with the spore killer phenotype across the *N. sitophila* genome. (*B*) A single locus (*A*, red arrow) on chromosome 6 shows complete association between genotype and phenotype. (*C*) The locus (*sk1c1*) contains 25 variable sites with complete association spanning 1,933 bp and is contained within a highly diverged 23-kbp region which is found in 21 of 25 *Sk-1* strains. (*D*) In sensitive *N. sitophila* strains, *sk1c1* contains a homolog to the *N. crassa* gene *NCU09865*, but in *Sk-1* killer strains this gene is missing the first 375 bp, and it is instead replaced with 1.2 kbp of new sequence, here visible as white nonaligning regions between the pale blue aligning regions. The region of complete association is marked in yellow. (*E*) A transcriptomic analysis of a sensitive x *Sk-1* cross reveals a single transcript (*Spk-1*) in the *sk1c1*^*k*^ region. The structure of the *Spk-1* transcript, including introns and RNA-seq coverage, is shown, together with the 5′ ends of the two genes flanking it. The RNA-seq coverage of *Spk-1* reaches a maximum of 374×. The transcript contains two candidate open reading frames: ORF2 corresponding to the remaining part of *NCU09865* and ORF1 showing homology to *N. crassa* gene *NCU01957*. A molecular dissection of the transcript shows that only ORF1 is necessary for spore killing (*SI Appendix*, Figs. S5 and S6). (*F*) No functional protein domains could be identified in *Spk-1* ORF1, but an InterPro scan predicted two transmembrane domains, a central cytoplasmic region, and two flanking noncytoplasmic regions. (*G*) Images of asci from dissected perithecia. When crossing a sensitive strain to an *Sk-1* strain, four viable black and four dead white spores are produced, indicating spore killing (1). In a cross between two sensitive *N. sitophila* strains, eight viable spores are formed in an ascus (2). When deleting the *sk1c1*^*k*^ locus (*Sk-1∆*) and crossing to a sensitive strain, killing is lost (3). When crossing *Sk-1∆* to another *Sk-1* strain, spore killing is observed, indicating loss of resistance to spore killing (4). When inserting *sk1c1*^*k*^ at the same locus in a sensitive strain (*9865∆:::sk1c1*) and crossing it to a sensitive strain, killing is observed (5) and when crossed to *Sk-1*, killing is lost, indicating gain of resistance (6). 5941 and W1432 were used as *Sk-1* tester strains, and 4739 and W1446 were used as sensitive tester strains.

In order to identify the locus responsible for spore killing, we conducted a genome-wide association test. Illumina reads were mapped to the PacBio assembly of the killer strain W1434, single-nucleotide polymorphisms (SNPs) were called, and the association of each biallelic site to the killer phenotype was calculated using Fisher’s exact test (Fig. 2*A*). Only 25 SNPs covering a 2-kbp region on chromosome 6 showed a perfect association with the killer phenotype (Fig. 2 *B* and *C*). We call this region *sk1c1*, for *Sk-1 candidate 1*. A closer inspection revealed that in both killer and sensitive strains, the region contains a homolog to the *N. crassa* gene *NCU09865*. Sensitive strains carry the whole *NCU09865* sequence, but in the killer strains the allele is truncated and is missing the first half of the open reading frame (ORF); in its stead there is ∼1 kbp of new sequence (Fig. 2*D*). *NCU09865* is of unknown function in *N. crassa*, but it is predicted to contain a methyltransferase domain and is most highly expressed during sexual development (30). The methyltransferase domain is present in the sensitive *N. sitophila* allele, but is partially missing in the *Sk-1* allele.

### Confirmation of the Killing and Resistance Activity of *sk1c1*^*k*^.

We tested if the killer *sk1c1* allele (*sk1c1*^*k*^) is responsible for spore killing using three different strategies. First, we tested if it showed distorted segregation. A cross between a killer strain (W1446) and a sensitive strain (W1426) was performed and 46 sexual spores were picked and germinated separately. Segregation of the killer allele was assessed by PCR, with primers detecting a size polymorphism at *sk1c1*. All 46 spores carried the killer allele, showing complete segregation distortion.

We then generated a deletion mutant of the locus by deleting 2.8 kbp surrounding *sk1c1*^*k*^ in strain W1434 (*SI Appendix*, Fig. S3). When the deletion mutant was crossed to sensitive strains no killing was observed, and when it was crossed to killer strains killing was detected (Fig. 2*G*). These results show that *sk1c1*^*k*^ is necessary both for killing sensitive strains and for resistance to killing from other *Sk-1* strains, and presumably itself.

Finally, we inserted *sk1c1*^*k*^ at the same locus in a sensitive *N. sitophila* strain (W1426), by replacing *NCU09865*. When we crossed this knockin strain to a sensitive strain (5941), spore killing was again detected (Fig. 2*G*), and when we crossed it to an *Sk-1* strain (W1434), killing was not observed, indicating that *sk1c1*^*k*^ is sufficient for both spore killing and for resistance. These results are consistent with a poison–antidote mechanism for *Sk-1*, where the presence of a resistance factor at *sk1c1*^*k*^ can rescue any strain from killing, independent of genetic background.

### An ORF with Homology to *NCU01957* Is Generating the Causative Agent for Spore Killing.

We generated transcriptomic data from W1434 (*Sk-1*), 5940 (sensitive), and a cross between the two strains. We identified a 1,450-bp transcript containing two introns at *sk1c1*^*k*^ in the data from W1434 and from the cross (Fig. 2*E* and *SI Appendix*, Fig. S4). This result indicates expression of this transcript both during sexual development and vegetative growth. The two longest predicted ORFs are just over 400 bp, one of which corresponds to the *NCU09865* fragment (ORF2) and the other which shows homology to the *N. crassa* gene *NCU01957* (ORF1). Furthermore, in *Neurospora*, A-to-I RNA editing is active during sexual development and ascospore formation (30). The transcript contains a single edited site, which converts a tyrosine in ORF1 to a cysteine.

In order to determine which ORF causes spore killing, or if both are necessary, we molecularly dissected the transcript. First, we performed RT-PCR using several primer pairs within the transcript, results of which support the existence of a single 1,450-bp locus-spanning transcript with two introns (*SI Appendix*, Fig. S5). Second, we generated deletion mutants of each ORF (*SI Appendix*, Figs. S5 and S6). The deletion mutant of ORF1 matched the expected phenotype of a sensitive strain: It showed loss of killing when crossed to a sensitive strain and loss of resistance when crossed to a killer strain. In contrast, the deletion mutant of ORF2 showed loss of killing but no loss of resistance.

We then generated mutants where the start codon (ATG) of each ORF was changed to a leucine (TTG) (*SI Appendix*, Fig. S7). Here we observed loss of killing and resistance in the ORF1 mutant (*SI Appendix*, Fig. S7 *B* and *E*) but loss of neither in the ORF2 mutant (*SI Appendix*, Fig. S7 *C* and *F*). We also generated an ORF2 frameshift mutant by inserting a guanine nucleotide immediately after the predicted start codon. Like the ORF2 ATG-to-TTG mutation, the ATG-to-ATGG mutation altered neither spore killing nor resistance to spore killing (*SI Appendix*, Fig. S7 *H* and *K*).

Finally, we created a mutant version of ORF2 called ORF2[mut5]. This sequence contains five single-nucleotide variants, nine single-nucleotide insertions, seven single-nucleotide deletions, and a 3-nt-long deletion (*SI Appendix*, Fig. S7*M*). The goal of ORF2[mut5] was to disrupt any and/or all coding sequences within ORF2. Interestingly, while ORF2[mut5] kills spores, it does so with reduced efficiency (*SI Appendix*, Fig. S7*I* and Table S3). In contrast, ORF2[mut5] appears to be fully efficient with respect to resistance to spore killing (*SI Appendix*, Fig. S7*L*).

Our results (summarized in *SI Appendix*, Table S4) suggest that a single gene exists at the *sk1c1*^*k*^ locus, herein referred to as *Spore killer-1* (*Spk-1*, the gene name starting with an uppercase letter because of its dominant phenotype). A single transcript is produced from *Spk-1* and a protein with 134 amino acids is translated from the *Spk-1* messenger RNA.

### *Spk-1* Has Many Different Homologs in *Neurospora*.

The SPK-1 protein contains no predicted functional domains, but an InterPro scan identifies two transmembrane domains, a central cytosolic region and two noncytosolic flanks (Fig. 2*F*). Searching the National Center for Biotechnology Information (NCBI) nonredundant protein database and FungiDB identifies no similar sequences outside of *Neurospora* but several hits in *N. crassa* and *Neurospora tetrasperma*. Two *N. crassa* genes show significant sequence identity (*SI Appendix*, Fig. S8), *NCU01957* (35% amino acid identity) and *NCU16600* (32% amino acid identity). *NCU01957* contains a plasma membrane ATPase domain not found in SPK-1 and mutants are sterile (31). No information on *NCU16600* function is available, but it is highly up-regulated late in perithecial development, when ascospores start to be delimited (32). *Spk-1* also does not share any detectable homology with any of the other known spore killer genes in *Neurospora*, *Podospora*, or *Schizosaccharomyces*.

We searched genome assemblies for 31 *Neurospora* strains from seven different species (*N. crassa*, *Neurospora discreta*, *N. hispaniola*, *N. intermedia*, *Neurospora metzenbergii*, *N. sitophila*, and *N. tetrasperma*; *SI Appendix*, Table S5) for homologous sequences of *Spk-1* using tblastn; 115 hits over 100 amino acids were identified (including *NCU01957* and *NCU16600*) with amino acid sequence identities ranging from 24 to 98.5% (*SI Appendix*, Fig. S9). It is not clear to what extent these hits represent actual genes, but in most cases they correspond to potential ORFs of approximately equal or greater length than *Spk-1*.

### *sk1c1*^*k*^ Shows Signals of Introgression from *N. hispaniola*.

**Fig. 3.**
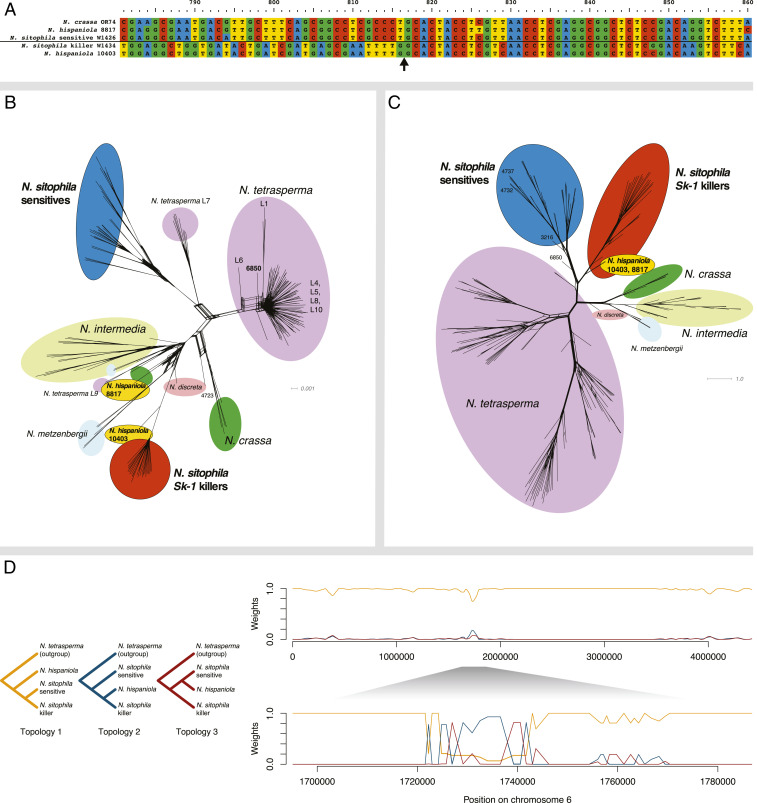
*Spk-1* is introgressed into *N. sitophila* from *N. hispaniola*. (*A*) Alignment of the *NCU09865* segment. *N. crassa* OR74, *N. hispaniola* 8817, and sensitive *N. sitophila* W1426 all carry the complete gene, but *Sk-1 N. sitophila* W1434 and *N. hispaniola* 10403 are both missing the first 375 bp of the coding region. The black arrow marks the shared breakpoint of the deletion. (*B*) Phylogenetic network of the *NCU09865* fragment remaining in *Spk-1*. *N. sitophila Sk-1* and sensitives form two distinct clades. *N. hispaniola* 10403 groups with *Sk-1*, whereas *N. hispaniola* 8817 groups with neither. (*C*) Phylogenetic network of the concatenated sequences of the two genes flanking *NCU09865*: *NCU09864* and *NCU09866*. *Sk-1* and sensitive *N. sitophila* still form distinct clades, but here both *N. hispaniola* strains group with *Sk-1*. (*D*) Twisst plots showing signals of introgression from *N. hispaniola* into either *Sk-1* or sensitive strains of *N. sitophila*. The plot is generated by inferring phylogenetic trees in sliding windows containing 50 variable sites, then sampling subtrees with a strain from each clade from the tree, and finally calculating the fraction of all subtrees that support each topology. (*D*, *Left*) The three possible phylogenetic topologies. (*D*, *Right Top*) Distribution of topologies across chromosome 6. (*D*, *Right Bottom*) The region surrounding *sk1c1*. In the *sk1c1* region, the dominant topology groups *Sk-1* strains with *N. hispaniola*, which is consistent with an introgressive scenario.

The homologous sequence that shows the highest sequence identity to *Spk-1* (98.5% amino acid identity) is found in *N. hispaniola* strain 10403 (33). This sequence is not only highly similar to *Spk-1* but it also truncates *NCU09865* at the same site (Fig. 3*A* and *SI Appendix*, Fig. S10). A phylogenetic analysis of the *NCU09865* fragment present in 10403 and the *Sk-1* killers, including alleles from several different species of *Neurospora*, reveals that all *Sk-1* killers group with 10403 but are clearly divergent from all sensitive *N. sitophila* alleles (Fig. 3*B*).

The two genes flanking *Spk-1* are located in the larger region that is fixed in 21 out of 25 killer strains and a phylogenetic analysis of these reveals that the *Sk-1* alleles are also most similar to alleles found in *N. hispaniola* (Fig. 3*C*). These sequences cluster not only with sequences from strain 10403 but also with *N. hispaniola* strain 8817, which does not carry *Spk-1* or the truncated *NCU09865* sequence. The *N. sitophila* killers and the two *N. hispaniola* strains form a monophyletic group clearly separated from the sensitive *N. sitophila* strains. A phylogenetic analysis based on SNP data in sliding windows across the genome also identifies the *sk1c1*^*k*^ allele as a candidate for introgression from *N. hispaniola* (Fig. 3*D*), but on a genome-wide level there is only evidence for very limited gene flow between the two species (*SI Appendix*, Figs. S11 and S12).

We interpret these results as suggesting that the killer locus has been introgressed into *N. sitophila* from *N. hispaniola*. However, we have a limited sample of *N. hispaniola* strains in our dataset and thus we cannot exclude the possibility that the source is another unknown species, or an unsampled and diverged population of *N. sitophila*.

### *N. sitophila* Is Split into Three Major Clades.

**Fig. 4.**
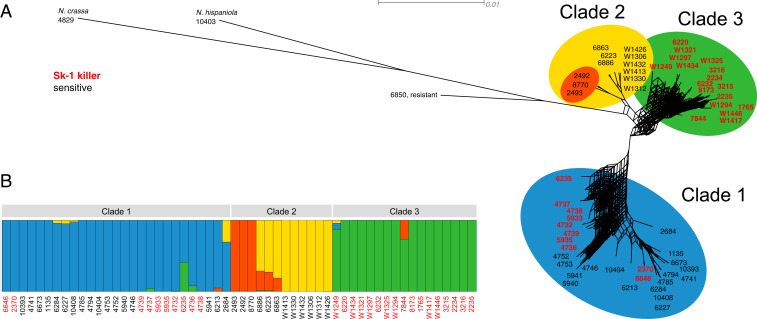
Population structure of *N. sitophila*. (*A*) A maximum-likelihood phylogeny based on whole-genome SNP data indicates that *N. sitophila* has clear population structure and can be split into three clades (clades 1, 2, and 3). Both *Sk-1* and sensitive strains can be found in clade 1, whereas clade 2 is fixed for sensitives and clade 3 is fixed for *Sk-1*, even though both clades 2 and 3 can be found at the same sampling sites. (*B*) An admixture analysis of *N. sitophila* at *K* = 4 further splits clade 2 and shows limited signal for gene flow between the clades.

A phylogenetic analysis of whole-genome SNP data reveals clear population structure within *N. sitophila* and the investigated strains were split into three major clades (Fig. 4*A*), from now on referred to as clades 1, 2, and 3. Clade 1 has a global distribution with strains from Asia, Oceania, Africa, the Caribbean, and Central and South America. Clade 2 contains strains from Asia, Africa, and Europe, and clade 3 contains strains from Europe, Australia, and the continental United States. All strains from Tahiti, both *Sk-1* and sensitives, are placed in clade 1, whereas all sensitive strains from Italy are placed in clade 2 and all *Sk-1* strains from Italy are placed in clade 3. In fact, clade 2 is completely fixed for the sensitive genotype and clade 3 for *Sk-1*. We only find evidence for limited gene flow between the three clades (Fig. 4*B*), even though strains from clades 2 and 3 were sampled at the same site and at the same time in Italy (27). These strains show no reduced fertility in laboratory crosses, but nevertheless reproduction between strains of these clades appears to be rare in nature. Additionally, one strain of *N. sitophila* (6850) has been classified as resistant to *Sk-1* (28), but based on our analysis it shows comparatively strong differentiation from all other *N. sitophila* strains, and it may be classified as a different species.

### Spore Killing Can Be Suppressed through RNAi.

**Fig. 5.**
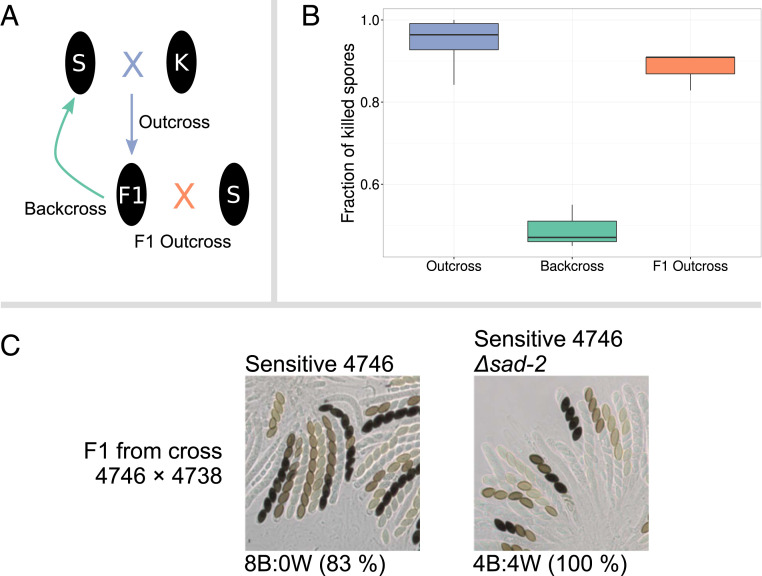
MSUD can suppress spore killing. (*A*) Crossing design to evaluate the strength of spore killing. S represents the sensitive parent 4746; K represents the *Sk-1* parent 4738. The F1 outcross was conducted to strain W1426. (*B*) Plot showing the fraction of asci exhibiting spore killing. (*C*) The effect of the *sad-2* deletion on the ability of backcrossed strains to kill. In a cross between a sensitive strain from Tahiti (4746) and a killer F1 from a cross between that strain and a killer strain (4738), killing is strongly reduced. Deleting the *sad-2* gene inactivates MSUD, and in a cross between 4746 Δ*sad-2* and the killer F1, spore killing is restored to normal levels. This indicates that MSUD is responsible for suppressing spore killing in inbred crosses with a genetic background from Tahiti. This pattern is not seen if the sensitive strain comes from Italy.

We wanted to investigate if the differences in frequency of *Sk-1* between the clades could be influenced by the genetic background. We therefore set out to introgress *Sk-1* alleles from Italy and Tahiti into sensitive backgrounds from both regions and create isogenic lines by multiple rounds of backcrossing (Fig. 5*A*). In the first round of crosses, we observed normal levels of spore killing, but when backcrossing first generation offspring (F1) individuals with a Tahitian sensitive parent to that same sensitive parent, we saw a strong reduction in the number of four-spored asci (Fig. 5*B*). Furthermore, after three generations of backcrossing, all lines generated from Tahitian sensitive parents had either completely lost spore killing or had a large reduction in spore killing (*SI Appendix*, Fig. S13). For the Italian sensitive lines, all lines displayed normal spore-killing phenotypes. This pattern could be explained by *Spk-1* becoming unlinked from a modifying locus that improves killing efficiency, but when the Tahitian F1 individuals were outcrossed to another sensitive strain from either location, the number of four-spored asci was close to normal levels. This pattern suggests that the loss of spore killing is not caused by unlinking a modifier locus but instead that it is an effect of increased relatedness between the killer and sensitive strains.

MSUD is a genome defense mechanism identified in *Neurospora* that operates through the RNAi machinery to posttranscriptionally silence genes that are unpaired during meiosis (34). Theoretically, MSUD should be capable of targeting and inactivating *Spk-1* as it is partially unpaired in a killer–sensitive cross; however, MSUD activity has not been evaluated in *N. sitophila*. MSUD has been shown to have higher efficiency in inbred lines of *N. crassa* (35), and we hypothesized that loss of killing could be driven by an increased MSUD activity caused by increased relatedness of the killer and sensitive strains. To test this hypothesis, we generated a strain that lacks functional MSUD by deleting *sad-2* (36) in the sensitive Tahitian strain 4746. When *Sk-1*–proficient offspring of 4746 were backcrossed to the *sad-2*–deficient parent, close to 100% four-spored asci was observed (Fig. 5*C* and *SI Appendix*, Figs. S13 and S14), indicating that MSUD is indeed responsible for the loss of killing.

## Discussion

We have identified the gene responsible for the *Sk-1* spore killer phenotype in *N. sitophila*. A 1,450-bp transcript generates a single protein product of no more than 134 amino acids which is capable of both killing sensitive sibling spores and of protecting the spores producing the protein from self-killing. We have found no obvious mechanism behind either the killing or resistance phenotypes, but the gene carries predicted transmembrane domains, presenting the possibility that it could disrupt membrane integrity, as has been observed in some bacterial toxin–antitoxin systems (37, 38). On the other hand, the *wtf* genes in *Schizosaccharomyces* were initially also predicted to contain transmembrane domains (13), but recent work has shown that these are hydrophobic regions which are important for forming large toxic protein aggregates essential for spore killing (39), and a similar mechanism might be at play here as well.

The *Spk-1* transcript is a chimera of the 3′ end of *NCU09865* and a sequence similar to *NCU01957*. While the remaining fragment of *NCU09865* including ORF2 appears to be well-preserved, our results indicate that only ORF1 with homology to *NCU01957* is translated. Even so, the remains of *NCU09865* still play a role, as deleting or heavily mutating ORF2 affects the killing function. The mechanistic basis for loss of killing in *ORF2*Δ but not *ORF2[ATG-to-TTG]* or *ORF2[ATG-to-ATGG]* start-codon mutants is unclear. It could be that the *Spk-1* 3′ untranslated region (UTR) contains a regulatory element that is required for killing but not resistance. This idea is consistent with our findings for *ORF2[mut5]*, which kills spores with decreased efficiency. The decreased efficiency suggests that one or more of the mutations in *ORF2[mut5]* lies within an important regulatory sequence of the 3′ UTR. Furthermore, a single site in the *Spk-1* transcript experiences A-to-I RNA editing, which converts a tyrosine to a cytosine in 26% of all transcripts. Further work is necessary to determine if it is important for either killing or resistance, but one intriguing hypothesis is that it is RNA editing that makes it possible for the single *Spk-1* gene to perform both functions by generating two different proteins. This question could be addressed by for instance constitutively expressing edited and unedited products in vegetative and sexual tissues.

*Spk-1* shows no homology to any known spore killer gene and, compared with the *wtf* genes in *Schizosaccharomyces* and the *Spok* genes in *Podospora*, *Spk-1* is significantly smaller (9, 10, 12, 13). On the other hand, ORF1 appears to belong to a family of highly diverse *Neurospora*-specific genomic sequences. It is still unclear to what extent these sequences are protein-coding, but among them we found two annotated genes in the *N. crassa* reference genome. *NCU01957* is located very close to the mating-type locus of *N. crassa*, but is not found at this location or elsewhere in most other investigated *Neurospora* genomes. Exceptions are *N. hispaniola*, where it is found at the same locus, and *N. sitophila*, where a highly similar gene is found at a distance of ∼20 kbp. *NCU01957* is of unknown function, but a strain carrying a single-amino acid mutation displayed both stunted vegetative growth and total sterility (31). *NCU16600* is also of unknown function, but it is up-regulated during ascosporogenesis (32). The highly diverse set of homologous sequences is reminiscent of the *wtf* genes in *Schizosaccharomyces*, with complex phylogenetic patterns and extensive presence–absence polymorphisms (14). Further work is necessary to determine if these are genes with a regular function for normal sexual development, or if they could be selfish genetic elements that have either gone to fixation or are controlled by some suppressing mechanism.

The *sk1c1*^*k*^ allele found in *N. sitophila Sk-1* strains is highly diverged from sensitive *N. sitophila* strains and together with the flanking genes shows greater similarity to the closely related species *N. hispaniola*. Strain 10403 carries a copy of *Spk-1* that shows both high sequence and structural similarity to the one found in *Sk-1* strains, suggesting that it may also be able to cause spore killing. Only four strains of *N. hispaniola* have ever been collected (33), and we have not been able to observe spore killing when crossing these to each other or in hybrid crosses to *N. tetrasperma*. This could mean that *Spk-1* in 10403 is no longer functional, but it can also indicate that the strains available carry an unknown resistance factor and further sampling would be necessary to assess whether *Spk-1* is actively driving within the species.

While *sk1c1*^*k*^ appears to be introgressed from *N. hispaniola*, there is little sign of introgression elsewhere in the genome. *N. sitophila* and *N. hispaniola* show high levels of reproductive isolation with each other and we have so far not observed viable offspring in any crosses between the species. While selfish genetic elements such as meiotic drivers have often been argued to contribute to restricting gene flow between species (17, 18), here we see the opposite result, where meiotic drive instead may have facilitated a limited exchange of genetic material. Similar results have been observed in *D. simulans* and *Drosophila mauritiania* where the Winters sex ratio element has crossed between species despite strong reproductive barriers (21). At this time, we cannot exclude that *Sk-1* entered *N. sitophila* through a yet-undiscovered species or population of *Neurospora* that shows greater sexual compatibility with both *N. sitophila* and *N. hispaniola*. We also do not know if *Spk-1* evolved in *N. hispaniola* or if it originated in another species and has spread within *Neurospora* through multiple introgressive steps.

Using whole-genome SNP data, we can show that *N. sitophila* is split into three clades which show remarkably different patterns of spore killing. While 15% of all *N. sitophila* strains carry the *Sk-1* element, these are not evenly distributed within the clades. Clade 3 is fixed for *Sk-1*, clade 2 is fixed for sensitives, and clade 1 contains both genotypes. This population structure is especially puzzling as strains from clades 2 and 3 were sampled at the same location at the same time and show no signs of reduced fertility when crossed under laboratory conditions but still show only very limited signatures of gene flow. Some mechanism must prevent individuals from these two clades from crossing in nature, but unfortunately we currently know too little about the ecology and life cycle of *N. sitophila* to have a good understanding of what this might be.

In clade 1, we find both *Sk-1* and sensitive strains. It is unclear if *Sk-1* has recently been introduced into the clade and is on its way to fixation or if it is maintained as a stable polymorphism due to unknown fitness costs of carrying the spore killer element. One tantalizing suggestion is that the *Sk-1* polymorphism can be explained by the observed suppression through MSUD. While we only observe strong suppression in highly isogenic crosses, we do not know how common such crosses are in nature. If inbreeding is a common pattern in *N. sitophila* and MSUD is more efficient between inbred strains, MSUD may be sufficient enough to keep *Sk-1* at a low frequency. It is also possible that MSUD is more efficient under natural growing conditions where environmental factors may promote a higher activity.

RNAi has been shown to be essential for suppressing sex-linked meiotic drive in *D. simulans* and is suspected to be involved in other *Drosophila* species as well (16, 40, 41). In *D. simulans* the sex ratio driver *Dox* is suppressed by two genes named *Nmy* and *Tmy* (16, 42, 43). Both genes are partial duplicates of *Dox* and generate small RNAs which silence *Dox* expression and prevent inactivation of Y-bearing sperm. The production of small RNAs has also been observed from duplicated genes that are suspected to cause sex ratio distortion in *Drosophila miranda* (41).

Here we show that the RNAi-based MSUD system can act as a suppressor of spore killing in highly isogenic crosses. Transcriptional silencing through MSUD is not dependent on gene duplications but can act directly on genes that are unpaired at meiosis. Silencing through gene duplication may actually not be possible in *Neurospora*, as duplicated sequences are targeted and mutated by the RIP (repeat induced point mutations) system, which will introduce high levels of C-to-T mutations in larger sequences found at least twice in a genome (44). Both MSUD and RIP are thought to defend against selfish genetic elements such as transposable elements, and MSUD should at least in theory be capable of defending against invading meiotic drivers that are sufficiently divergent to not be able to pair at meiosis (45). It is interesting to note that both *Spk-1* in *N. sitophila* and *rfk-1*, which is the causative factor behind the *Sk-2* spore killer element in *N. intermedia* (26), manage to evade MSUD in most crosses. In the case of *rfk-1*, the small size together with proximity to pairing DNA appears to protect it from being silenced (26), and in the case of *Spk-1* a potentially higher MSUD efficiency in isogenic crosses is enough to tip the balance against the spore killer gene. We can hypothesize that MSUD is often a sufficient defense against most KMDs which try to invade a population of *Neurospora*, and the only ones that are able to persist or go to fixation are the rare cases that have some feature that allows them to evade genome defense mechanisms such as MSUD.

Most of the killer meiotic drive elements that were initially discovered and which have been studied in greatest detail are large multigene systems found in regions of suppressed recombination (5–8). It has sometimes even been stated that meiotic drive can only be caused by the interaction of multiple loci and that the association with regions of low recombination is a defining characteristic of the phenomenon. *Spk-1* joins a growing number of recently discovered single-gene KMDs which contradict this view. In fungi, four out of five known meiotic drive gene families are caused by single genes which can cause drive on their own (9, 11, 13, 26). All of these gene families appear to have evolved independently, suggesting that there is ample potential for de novo evolution of single-gene KMDs. But if this is the case, why have we not found more of these systems? Single genes are less likely to be tightly linked to deleterious mutations, which could make them more likely to go to fixation and become invisible in crosses. They are also less likely to be tightly linked to visible phenotypes that would allow easy detection. Although spore-killing phenotypes can be relatively easy to detect, the genomic techniques used here can also be used to detect segregation distortion in the absence of visible phenotypes. Indeed, we expect that future work combining genomic techniques, controlled crosses, and sampling of more nonmodel organisms will unmask a vast diversity of cryptic meiotic drivers and shed new light on meiotic drive as a major factor in the evolution of species.

## Methods

All natural isolates used in this study are listed in *SI Appendix*, Table S2. Strains were ordered from the Fungal Genetics Stock Center (46) or provided by D.J.J. (27). We verified the phenotype of all strains by crossing them to both *Sk-1* and sensitive tester strains and observing patterns of dead and living spores at 500× magnification. This method was also used to determine the efficiency of spore killing among the introgressed lines generated between Tahitian and Italian strains for the MSUD analysis.

Whole-genome paired-end Illumina HiSeq sequencing data were collected from 56 *N. sitophila* strains (*SI Appendix*, Table S2), and long-read PacBio data were collected from 4 strains (*SI Appendix*, Table S1). Transcriptomic data were also collected by paired-end Illumina sequencing of RNA from vegetative tissue of the *Sk-1* strain W1434 and the sensitive strain 5940, and sexual tissue was obtained from a cross between the two. We assembled the PacBio data de novo using HGAP 3.0 (47), and checked completeness and synteny by aligning the assemblies to the *N. crassa* reference genome. We then called variants by mapping the Illumina reads to the PacBio assembly of strain W1434, and calculated the association of each SNP with the killing phenotype using Fisher’s exact test. A transcriptome was also assembled for W1434 by mapping RNA-sequencing (RNA-seq) reads to the PacBio assembly with STAR (48) and calling transcripts using Cufflinks (49).

We tested whether *sk1c1* showed segregation distortion by evaluating 46 progeny from a cross between strains W1426 and W1446 for the abundance of each parental allele. We developed a PCR protocol that produces an ∼900-bp product in W1446 (killer locus) and an ∼600-bp product in W1426 (sensitive locus) and assessed the genotype of the progeny with gel electrophoresis. All genetic transformations were performed by electroporation and gene-deletion and transgene-insertion vectors were constructed by double-joint PCR.

Homologs of *Spk-1* were identified with BLASTp against the NCBI nonredundant protein database, FungiDB, and 31 additional high-quality *Neurospora* assemblies. Hits longer than 100 bp were extracted and a phylogeny was inferred using IQ-TREE (50). We tested the hypothesis of introgression by extracting the fragment of *NCU09865* found in *Spk-1* from 177 *Neurospora* assemblies, as well as the two neighboring genes *NCU09864* and *NCU09866*, and generating phylogenies using RAxML (51). A whole-genome phylogeny of all 56 *N. sitophila* genomes was also generated from SNP data with RAxML. We searched for a genome-wide signal of introgression using a sliding window phylogenetic approach with Twisst (52), and evaluated introgression and between-clade gene flow with ADMIXTURE (53).

Full methods are included in *SI Appendix*, *Methods*.

## Supplementary Material

Supplementary File

## Data Availability

Raw reads reported in this article have been deposited in the Sequence Read Archive at the NCBI (under PRJNA649678). Genome assemblies, genome annotations, SNP data in variant call format, and alignments are available at FigShare (https://doi.org/10.6084/m9.figshare.c.5093585.v1) (54). All natural *N. sitophila* isolates used in this study that were previously not publicly available, together with key mutants generated for this study, have been deposited at the Fungal Genetics Stock Center. Scripts used for data analysis are available at https://github.com/johannessonlab/sitophila_spore_killer/.
