# Supplementary material for: An introgressed gene causes meiotic drive in Neurospora sitophila
Source: Proc Natl Acad Sci U S A. 2021 Apr 19;118(17):e2026605118. doi: 10.1073/pnas.2026605118 (PMC8092558; doi:10.1073/pnas.2026605118)
Supplement: Supplementary File [file pnas.2026605118.sapp.pdf]

PNAS
www.pnas.org

Supplementary Information for
An introgressed gene causes meiotic drive in *Neurospora sitophila*

Jesper Svedberg, Aaron A. Vogan, Nicholas A. Rhoades, Dilini Sarmarajeewa, David J. Jacobson, Martin Lascoux, Thomas M. Hammond, Hanna Johannesson

Corresponding authors: Jesper Svedberg, Hanna Johannesson
Email: jsvedberg@gmail.com, hanna.johannesson@ebc.uu.se

This PDF file includes:

Supplementary methods
SI References
Figures S1 to S14
Tables S1 to S9

Supplementary methods

Strains

All natural isolates used in this study are listed in Table S2. Strains were ordered from the Fungal Genetics Stock Center (FGSC, http://www.fgsc.net, (1)), except those whose names start with W (e.g., W1434), which were provided by D. Jacobson (2). Most strains were annotated as either *Sk-1* spore killers or sensitives, and we verified these phenotypes and tested the strains without annotation by crossing them to both *Sk-1* killer and sensitive tester strains carrying the *fluffy* mutation, and patterns of spore killing were then observed using a microscope. The tester strains are listed in Table S6. Strains used for molecular characterization are listed in Table S7.

Furthermore, we used the *N. crassa* OR74 genome (3), corrected for assembly errors discovered by (4), as well as a dataset of 92 *N. tetrasperma*, one *N. sitophila* and one *N. hispaniola* strain (5).

All natural isolates previously not available from FGSC, together with key mutants used during molecular characterization were deposited at FGSC (Table S8).

Culture conditions

For maintenance, strains were grown in ambient conditions on Vogel's medium (6). Crosses were conducted in liquid synthetic crossing media (SC) with filter paper and no added sucrose. Two different protocols were used to isolate and visualize ascospores. 1) Single ascospores were collected from the sides of culture tubes and plated onto 2% water agar or Vogel's medium. These plates were heat treated at 60°C for 60 minutes to induce germination. Germinating ascospores were excised from the water agar and transferred to Vogel's media for growth and storage. Spore killing was evaluated when ascospores could first be observed on the walls of the culture tubes, after which mature perithecia were removed, dissected and imaged under magnification. 2) Ascospores were collected from lids of crossing plates, suspended in sterile water, and incubated for at least one day at 4°C. Ascospore suspensions were then heated at 60°C for 30 minutes and then plated on Vogel's medium. Germlings were transferred to Vogel's medium in culture tubes for growth and storage. Spore killing was evaluated 14 days post-fertilization by dissecting perithecia and imaging perithecial contents under magnification.

Genome sequencing

Whole genome short-read data was collected from 56 *N. sitophila* strains (Table S2). Conidia were inoculated into 50 ml plastic culture tubes containing 10 ml of 3 % liquid malt extract medium. The tubes were incubated at 30 °C on a rotary shaker for 2–3 days and were then harvested by removing the mycelium from the culture tubes which was squeezed between filter paper to remove excess liquid. It was then cut into pieces and approximately 100 µg was allotted into 1.5 ml Eppendorf tubes, which were stored at −20 °C until extraction. Genomic DNA was extracted using the Fungal/Bacterial Microprep Kit (Zymo, www.zymo.com) and sent to the SNP&SEQ Technology Unit (Science for Life Laboratory, Uppsala, Sweden), where libraries were prepared and sequenced on an Illumina HiSeq 2500 system.

Four *N. sitophila* strains (Table S1) were sequenced using the PacBio RSII platform (Pacific Biosciences). The strains were cultured by inoculating 200 ml of 3% liquid malt extract medium in 500 ml Erlenmeyer flasks with conidia. The flasks were placed on a rotary shaker at 30 °C for 3–4 days. The cultures were harvested by removing the mycelium from the liquid, placing it on a filter paper, which was folded between several layers of tissue paper and pressed to remove excess liquid. The mycelium was cut into smaller pieces and approximately 1 g was allotted into 2 ml tubes with screw-on caps. These tubes were then stored at −20 °C. To extract genomic DNA, two tubes of each strain were freeze-dried overnight and macerated using a TissueLyzer II bead-beater (Qiagen). Two 2 mm metal beads were placed in each tube, which were then shaken for 20 s at 25 Hz. If a tube contained large pieces of freeze-dried mycelium, it was shaken for another 10 s. This procedure was repeated until no large pieces remained. No tube was shaken for more than 40 s in total. DNA was finally extracted using Genomic Tip G-500 columns (Qiagen) and cleaned using the PowerClean DNA Clean-Up kit (MoBio Labs). Library preparation and sequencing was performed at the Uppsala Genome Center (Science for Life Laboratory, Uppsala, Sweden) using the C4 chemistry, P6 polymerase and four SMRT cells per strain (Pacific Biosciences).

Genome assembly

Raw PacBio sequence data was filtered using the SMRT Analysis package and assembled *de novo* using the HGAP 3.0 assembler (7) (Pacific Biosciences, https://github.com/PacificBiosciences/). Sequencing information and assembly statistics are shown in Table S1. The W1426 and W1434 assemblies were also annotated using MAKER (8), using protein and transcript sequences from *N. crassa* OR74 (3).

Raw Illumina HiSeq reads were assembled *de novo* using ABySS (9), with the following parameters: Kmer size = 64, bubble size = 3, seed length=200. Sequencing information and assembly statistics is shown in Table S2.

Whole genome alignment

Synteny and collinearity of the PacBio assemblies was investigated through whole genome alignment to the high-quality *N. crassa* OR74 reference genome (3) and to each other. The PacBio assemblies were aligned using MUMmer (10) with the parameters "nucmer –c 200 –b 2000". The alignments were visualised using MUMmer's plotting function mummerplot. Chromosome numbers were also assigned based on the corresponding *N. crassa* chromosome.

SNP calling

Raw Illumina HiSeq reads were cleaned from adapter contamination using CutAdapt (11) and trimmed using Trimmomatic (12). The reads were then mapped to the W1434 PacBio assembly with BWA (13). The resulting BAM file was deduplicated with picard (14), and complex regions were realigned with GATK IndelRealigner (15). SNPs were then called with GATK (first using -T HaplotypeCaller -bamWriterType CALLED_HAPLOTYPES -stand_emit_conf 10.0 -stand_call_conf 20.0 -gt_mode DISCOVERY -emitRefConfidence BP_RESOLUTION, then all VCF files were merged using -T GenotypeGVCF -sample_ploidy 1 -includeNonVariantSites). Sites with missing data and variants from regions called as repetitive with RepeatMasker were removed using VCFtools (16).

Genome wide association of variation to *Sk-1*

In order to identify the locus or loci responsible for spore killing in *Sk-1* strains, the association of each SNP to the killing phenotype was calculated by a custom Python script which performed Fisher's exact test for each variable site (available at https://github.com/johannessonlab/sitophila_spore_killer). The output was visualised with the python script manhattan-plot.py, which was downloaded from https://github.com/brentp/bio-playground/tree/master/plots/.

Characterization of the killer locus

The gene located at *sk1c1* was identified as a homolog to *NCU09865* by the MAKER genome annotation package (8), when annotating the sensitive strain W1426. The similarity of the truncated fragment in the killer strain W1434 to *NCU09865* was

confirmed using BLAST (17). Homologous sequences to *NCU09865* found in other closely related species had in many cases been annotated as having a methylase or methyltransferase domain. We confirmed this in *NCU09865* by using EBI's HMMer tool (18), which reports the presence of a Methyltransf_11 domain (PF08241.11).

Generating and analyzing transcriptomic data

Transcriptomic data was generated from vegetative tissue of *Sk-1* strain W1434, sensitive strain 5940, and from a sexual cross between both strains. Each strain was grown separately on solid Vogel's medium on petri dishes covered in cellophane for 2 days at 25 °C, under 12h:12h light–dark conditions. Hyphal tissue was then harvested from the surface of the cellophane with a sterile scalpel, and stored at -80°C. The sexual cross was performed on synthetic crossing medium (pH 6.5, 1.5% sucrose; (19)) with Wattman filter paper embedded into the medium, and covered with cellophane in 100 mm diameter petri dishes.

The harvested hyphal tissue was immediately frozen in liquid nitrogen and stored at 80˚C until RNA extraction. Next, 150 mg of the frozen tissue was ground together with liquid nitrogen and total RNA was extracted using the RNeasy Plant Mini Kit (Qiagen, Hilden, Germany). RNA quality was checked on the Agilent 2100 Bioanalyzer (Agilent Technologies, USA). All RNA samples were treated with DNaseI (Thermo Scientific) and sequencing libraries were prepared using the NEBNext Ultra Directional RNA Library Prep Kit for Illumina (New England Biolabs). mRNA was selected by purifying polyA+ transcripts (NEBNext Poly(A) mRNA Magnetic Isolation Module, New England Biolabs). Finally, the three paired-end libraries were sequenced with Illumina HiSeq 2500 at the SNP and SEQ Technology platform, generating 125 bp paired-end reads. The raw reads were trimmed using CutAdapt and Trimmomatic. They were then mapped to PacBio assemblies of the parental strains using STAR (20). In the case of the cross, the *sk1c1*^{k} region from W1434 was added as a separate contig to the 5941 genome assembly. Transcripts were finally called using cufflinks (21).

Phylogenomic analysis of *N. sitophila*

A whole genome phylogenetic analysis was performed with RAxML (22) using the SNPs called against the W1434 PacBio assembly, which had been converted into a fasta file containing all sites with GATK VariantsToTable and a custom Python script (availabe at https://github.com/johannessonlab/sitophila_spore_killer). RAxML was run with the following parameters: raxmlHPC-HYBRID-AVX -m GTRCAT -# 100 -f a. Phylogenies of each chromosome were generated the same way and phylogenetic

discordance was visualized by merging the trees into a Consensus Network with Splitstree (23).

Identification of SPK-1 homologs

We searched the NCBI non-redundant protein database and FungiDB's (24) protein database using blastp and default parameters. We also used tblastn (25) to locate homologous sequences to the SPK-1 protein in 31 *Neurospora* genome assemblies, most of which were high-quality assemblies based on PacBio sequencing data (Table S4). Hits longer than 100 aa were extracted, flanking sequence corresponding to 60 aa were added. Candidate start and stop codons then were manually identified and the sequences were trimmed. We aligned these sequences using MAFFT 7.407 (26) with default parameters and inferred a maximum likelihood phylogeny using IQ-TREE (27) with "parameters -st AA -m TEST -bb 1000 -wbt -alrt 1000".

Detecting introgression from *N. hispaniola* at *sk1c1*

Phylogenies of the *NCU09865* fragment located at $sk1c1^k$ and two neighbouring genes were also inferred. Homologous sequences to the *NCU09865* fragment were extracted from *de novo* assemblies of all *N. sitophila* strains analyzed in this project, together with 29 further *Neurospora* assemblies from (28) and 92 *N. tetrasperma*, *1 N. sitophila* and *1 N. hispaniola* assembly from (5), by using genBlastG (29) to extract the top hit to the *N. crassa NCU09865* protein sequence from each genome assembly. The sequences were aligned with MAFFT (26) and the alignment was manually trimmed using AliView (30). Finally, phylogenetic relationships were inferred using RAxML, with the same parameters as above. The two neighbouring genes were extracted in the same way, by extracting the top hits to *NCU09864* and *NCU098766*. These two alignments were then concatenated and analyzed with RAxML as above.

Detecting introgression from *N. hispaniola* across the genome

We used the program Twisst (31) to study how the phylogenetic signal varies across the genome, by following the pipeline described at https://github.com/simonhmartin/twisst. VCF files with SNPs called against W1434 were converted to the ".geno" file format used by Twisst, and local phylogenies were inferred in non-overlapping windows containing 50 SNPs using the Neighbour-joining algorithm of PhyML (32), with the following parameters: "phyml_sliding_windows.py -w 50 --windType sites --model GTR --genoFormat haplo". Twisst was then used to cluster local tree topologies with the parameters "run_twisst_parallel.py -T 4 --method

complete". Finally the output was plotted using the R script provided by the Twisst package.

Admixture analysis

Admixture between the different *N. sitophila* populations and from *N. hispaniola* was assessed using the software ADMIXTURE (33) using the W1434 SNP dataset and running the analysis from 2 to 8 ancestral populations.

PCR verification of segregation of *sk1c1*^{k}

Strains W1426 and W1446 were mated in liquid synthetic crossing media (SC) with filter paper and no added sucrose. Single ascospores were collected from the sides culture tubes and plated onto 2% water agar. These plates were heat treated at 60˚C for 1 hour to induce germination and kill any conidia or mycelia that may have been transferred along with the ascospores. Germinating ascospores were excised from water agar plates and transferred to Vogel's media from growth and storage. 46 progeny were obtained.

DNA was extracted using the Chelex 100 protocol (Bio-Rad). Primers were designed around a length polymorphism in the *sk1c1* region. The primers SK1C1F - ACCTCATCGTTCTGCAGCCCTCAT and SK1C1R - CTCCGAGCGAGGCTTGTGTGC produce a PCR product of ~900bp in the parental killer strain W1446 and a product of ~600bp in the parental sensitive strain W1426. The PCR protocol was as follows: initial denaturing at 98˚C for 30s, 30 cycles of 98˚C for 15s, 60˚C for 30s, 72˚C for 30s, and a final elongation step of 72˚C for 10m. Products were visualized on a 1% agarose gel.

Genetic transformation and strain construction

N. sitophila conidia were transformed by electroporation as described by Margolin et al (34). Homokaryotic transformants were isolated with the method of Ebbole and Sachs (35) or by screening single-conidium derived isolates. Gene deletion and transgene insertion vectors were constructed by double joint PCR (DJ-PCR) (36) using the primers listed in Table S9. The "left" and "right" DNA flanks for homologous recombination were amplified from genomic DNA of the transformation host. The "center" DNA fragments were amplified from various plasmids. Specifically, the center fragments for vectors v134, v178, v235, and v236 were amplified from pTH1256.1 (GenBank MH550659.1). The center fragment for v205a was amplified from pAY3.3. The center fragments for v205b, v205c, v205d, and v205e were

amplified from plasmids pNR139.5, pNR141.1, pNR202.1, and pNR203.1, respectively.

Plasmids pAY3.3, pNR139.5, pNR141.1, and pNR202.1 contain the *Spk-1* gene between the *Not*I and *Eco*RI sites of plasmid pTH1256.1. The *Spk-1* gene was amplified from strain W1434 with primers 1507 (5′ TTTTGCGGCCGCAGCATTTTCACCTTGGCCGTGAG 3′) and 1508 (5′ TTTTGAATTCGATATGGGGAACGGGATTGTGGA 3′).

Site-directed mutagenesis of the *Spk-1* sequence in pNR139.5, pNR141.1, and pNR202.1 was performed essentially as described by the Q5® Site-Directed Mutagenesis Kit (New England Biolabs). Specifically, for pNR139.5, the *Spk-1* ORF1 start codon was mutated to TTG with primers 1747 (5′ ATGTCGCAGATTGAACAACAC 3′) and 1748 (5′ TTACTATCATGTTAGAAAGGAG 3′). For pNR141.1, the *Spk-1* ORF2 start codon was mutated to TTG with primers 1751 (5′ ATACTGATCGTTGAGCGAATTTTGGC 3′) and 1752 (5′ CACCAGCCTCCAAACCAG 3′). For pNR202.1, the *Spk-1* ORF2 start codon was mutated to ATGG with primers 1892 (5′ GAGCGAATTTTGGCACTAC 3′) and 1893 (5′ CATCGATCAGTATCACCAG 3′). Plasmid pNR203.1 is identical to pAY3.3 except that the ORF2 sequence was replaced with ORF2$^{[mut5]}$ (Figure S7M). The ORF2$^{[mut5]}$ sequence was constructed by gene synthesis (gBlock®, Integrated DNA technologies).

Verification of transformant phenotype

Strains were mated in liquid synthetic crossing media (SC) with filter paper and no added sucrose. Strains 5941 and W1432 were used as sensitive tester strains. Strains 4739 and W1446 were used as killer tester strains. These strains were inoculated into culture tubes and allowed to grow for 3 days. Conidia from knockout strains were then spread over these cultures and cultures were allowed to mate over a 2-week period. Once ascospores could first be observed on the walls of the culture tubes, mature perithecia were removed and dissected. Rosettes of ascospores were imaged at 200x magnification.

MSUD analysis

Four *Sk-1* strains and four sensitives strains were selected and crossed to each other in an all-by-all fashion to determine the effect of genomic background on spore killing. Strains 4746, 5940, 4738 (*Sk-1*), and 4739 (*Sk-1*) were selected to represent Tahiti, and strains W1426, W1312, W1325 (*Sk-1*), and W1446 (*Sk-1*) were selected from Italy. In all cases, ascospores were randomly selected to generate progeny, conidia were

harvested and used to fertilize the sensitive parent to generate backcrossed lines. For one cross each of Tahiti x Tahiti (*Sk-1*), Tahiti x Italy (*Sk-1*), Italy x Tahiti (*Sk-1*), and Italy x Italy (*Sk-1*) lines were constructed in triplicate to control for any laboratory effects.

To quantify the strength of the loss of spore killing, new crosses were conducted between all parental strains. Perithecia were harvested and dissected at maturity and asci were scored for spore killing as either: killing (4-spored), no killing (8-spored), or intermediate (4 - 8 -spored) by manual counts under a dissecting microscope. Loss of killing was confirmed independently in two separate laboratories. The ability of F1 strains to perform spore killing when outcrossed was evaluated by crossing F1 progeny of 4746 x 4738 to the Italian sensitive strain W1426.

References

1. K. McCluskey, A. Wiest, M. Plamann, The Fungal Genetics Stock Center: a repository for 50 years of fungal genetics research. *J. Biosci.* **35**, 119–126 (2010).
2. D. J. Jacobson, *et al.*, New findings of *Neurospora* in Europe and comparisons of diversity in temperate climates on continental scales. *Mycologia* **98**, 550–559 (2006).
3. J. E. Galagan, *et al.*, The genome sequence of the filamentous fungus *Neurospora crassa*. *Nature* **422**, 859–868 (2003).
4. J. M. Galazka, *et al.*, *Neurospora* chromosomes are organized by blocks of importin alpha-dependent heterochromatin that are largely independent of H3K9me3. *Genome Res.* **26**, 1069–1080 (2016).
5. P. Corcoran, *et al.*, Introgression maintains the genetic integrity of the mating-type determining chromosome of the fungus *Neurospora tetrasperma*. *Genome Res.* **26**, 486–498 (2016).
6. H. J. Vogel, A convenient growth medium for *Neurospora* (Medium N). *Microb. Genet Bull* **13**, 42–43 (1956).
7. C.-S. Chin, *et al.*, Nonhybrid, finished microbial genome assemblies from long-read SMRT sequencing data. *Nat. Methods* **10**, 563–569 (2013).
8. B. L. Cantarel, *et al.*, MAKER: An easy-to-use annotation pipeline designed for emerging model organism genomes. *Genome Res.* **18**, 188–196 (2008).
9. J. T. Simpson, *et al.*, ABySS: A parallel assembler for short read sequence data. *Genome Res.* **19**, 1117–1123 (2009).
10. S. Kurtz, *et al.*, Versatile and open software for comparing large genomes. *Genome Biol.* **5**, R12 (2004).
11. M. Martin, Cutadapt removes adapter sequences from high-throughput sequencing reads. *EMBnet.journal* **17**, 10–12 (2011).

12. A. M. Bolger, M. Lohse, B. Usadel, Trimmomatic: a flexible trimmer for Illumina sequence data. *Bioinformatics* **30**, 2114–2120 (2014).
13. H. Li, R. Durbin, Fast and accurate short read alignment with Burrows–Wheeler transform. *Bioinformatics* **25**, 1754–1760 (2009).
14. , Picard toolkit. *Broad Inst. GitHub Repos.* (2019).
15. A. McKenna, *et al.*, The Genome Analysis Toolkit: A MapReduce framework for analyzing next-generation DNA sequencing data. *Genome Res.* **20**, 1297–1303 (2010).
16. P. Danecek, *et al.*, The variant call format and VCFtools. *Bioinformatics* **27**, 2156–2158 (2011).
17. S. F. Altschul, W. Gish, W. Miller, E. W. Myers, D. J. Lipman, Basic local alignment search tool. *J. Mol. Biol.* **215**, 403–410 (1990).
18. R. D. Finn, *et al.*, HMMER web server: 2015 update. *Nucleic Acids Res.* **43**, W30–W38 (2015).
19. M. Westergaard, H. K. Mitchell, *Neurospora* V. A synthetic medium favoring sexual reproduction. *Am. J. Bot.* **34**, 573–577 (1947).
20. A. Dobin, *et al.*, STAR: ultrafast universal RNA-seq aligner. *Bioinformatics* **29**, 15–21 (2013).
21. C. Trapnell, *et al.*, Differential gene and transcript expression analysis of RNA-seq experiments with TopHat and Cufflinks. *Nat. Protoc.* **7**, 562–578 (2012).
22. A. Stamatakis, RAxML version 8: a tool for phylogenetic analysis and post-analysis of large phylogenies. *Bioinformatics* **30**, 1312–1313 (2014).
23. D. H. Huson, D. Bryant, Application of phylogenetic networks in evolutionary studies. *Mol. Biol. Evol.* **23**, 254–267 (2006).
24. J. E. Stajich, *et al.*, FungiDB: an integrated functional genomics database for fungi. *Nucleic Acids Res.* **40**, D675–D681 (2012).
25. E. M. Gertz, Y.-K. Yu, R. Agarwala, A. A. Schäffer, S. F. Altschul, Composition-based statistics and translated nucleotide searches: Improving the TBLASTN module of BLAST. *BMC Biol.* **4**, 41 (2006).
26. K. Katoh, D. M. Standley, MAFFT Multiple sequence alignment software version 7: Improvements in performance and usability. *Mol. Biol. Evol.* **30**, 772–780 (2013).
27. L.-T. Nguyen, H. A. Schmidt, A. von Haeseler, B. Q. Minh, IQ-TREE: A fast and effective stochastic algorithm for estimating maximum-likelihood phylogenies. *Mol. Biol. Evol.* **32**, 268–274 (2015).
28. J. Svedberg, *et al.*, Convergent evolution of complex genomic rearrangements in two fungal meiotic drive elements. *Nat. Commun.* **9**, 4242 (2018).
29. R. She, *et al.*, genBlastG: using BLAST searches to build homologous gene models. *Bioinformatics* **27**, 2141–2143 (2011).
30. A. Larsson, AliView: a fast and lightweight alignment viewer and editor for

large datasets. *Bioinformatics* **30**, 3276–3278 (2014).
31. S. H. Martin, S. M. V. Belleghem, Exploring evolutionary relationships across the genome using topology weighting. *Genetics* **206**, 429–438 (2017).
32. S. Guindon, *et al.*, New algorithms and methods to estimate maximum-likelihood phylogenies: Assessing the performance of PhyML 3.0. *Syst. Biol.* **59**, 307–321 (2010).
33. D. H. Alexander, J. Novembre, K. Lange, Fast model-based estimation of ancestry in unrelated individuals. *Genome Res.* **19**, 1655–1664 (2009).
34. B. Margolin, M. Freitag, E. Selker, Improved plasmids for gene targeting at the his-3 locus of *Neurospora crassa* by electroporation. *Fungal Genet. Rep.* **44**, 34–36 (1997).
35. D. Ebbole, M. S. Sachs, A rapid and simple method for isolation of *Neurospora crassa* homokaryons using microconidia (1990) (July 13, 2018).
36. J.-H. Yu, *et al.*, Double-joint PCR: a PCR-based molecular tool for gene manipulations in filamentous fungi. *Fungal Genet. Biol. FG B* **41**, 973–981 (2004).

Supplementary figures

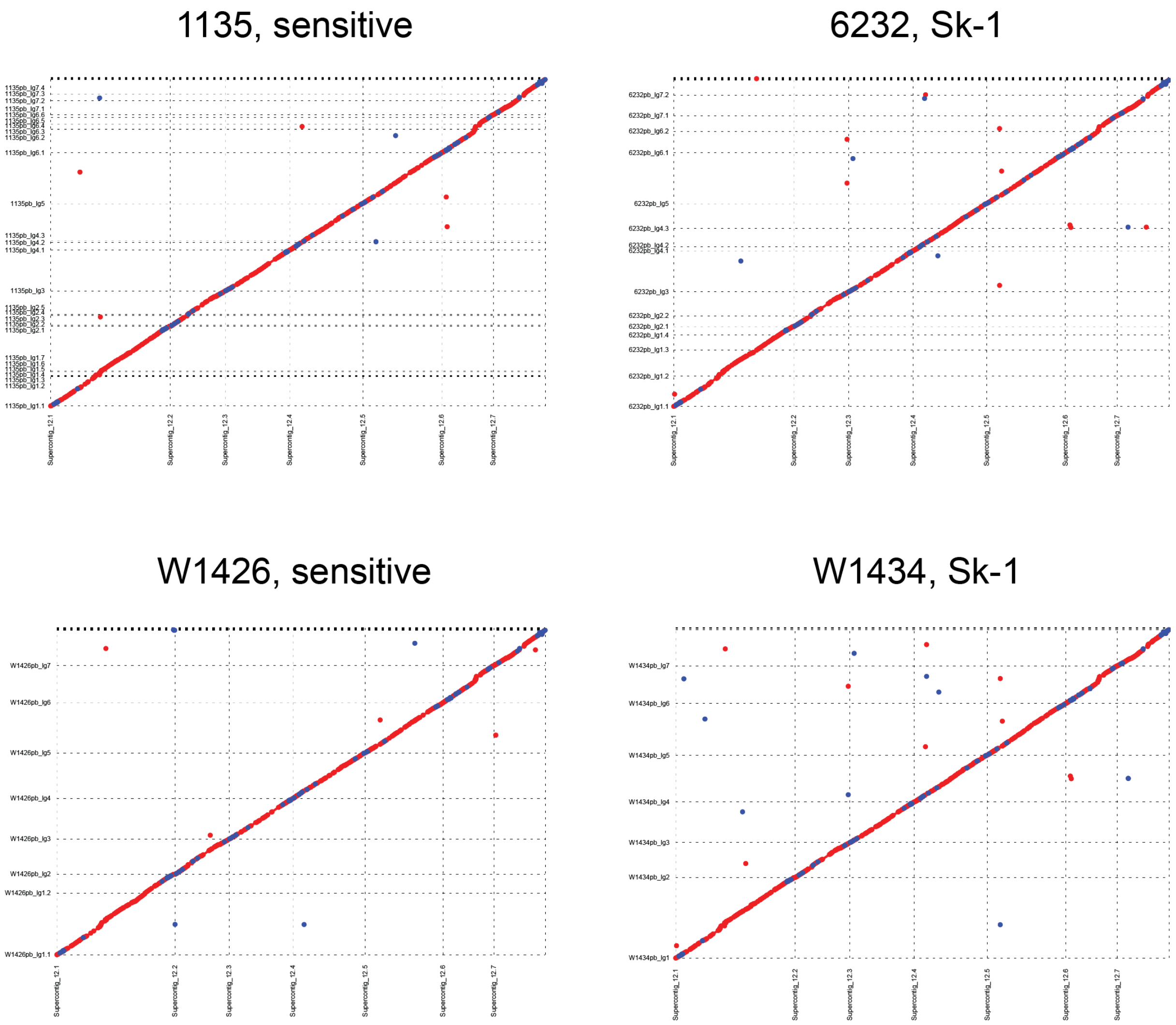

Figure S1: Dot plots of PacBio assemblies aligned to *N. crassa*

Dot plots showing whole genome alignment of the four PacBio assemblies of *N. sitophila* to the *N. crassa* OR74 reference assembly. PacBio contigs are plotted vertically, and *N. crassa* chromosomes horizontally.

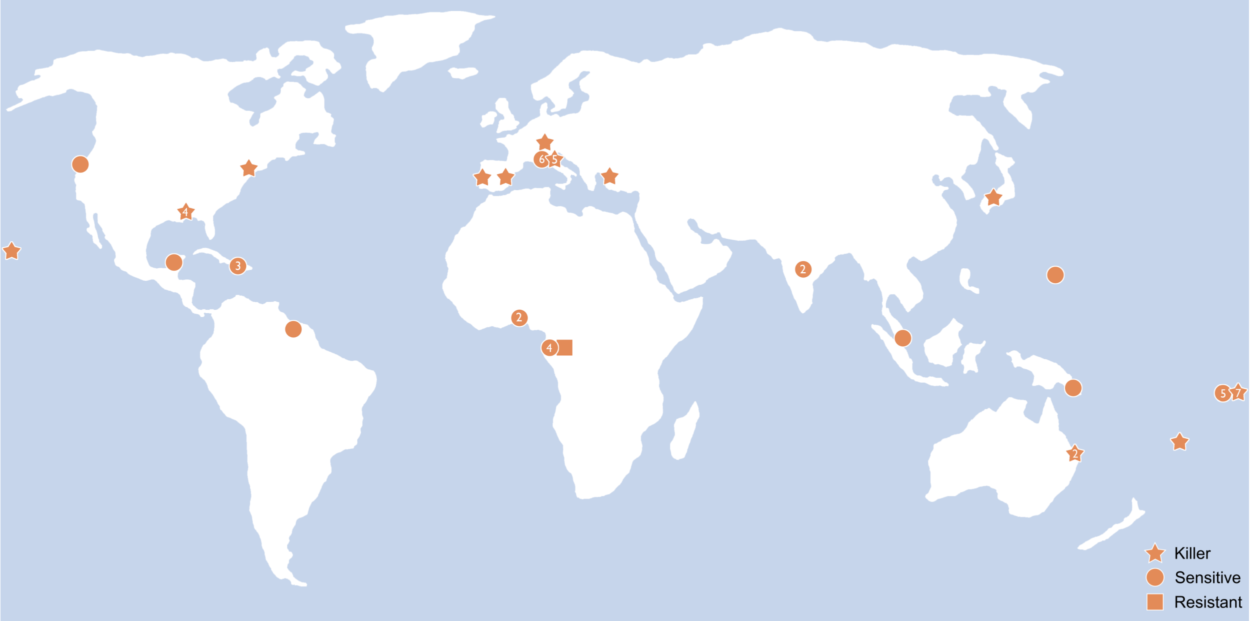

Figure S2: Map of samples

Map indicating sampling locations of all *N. sitophila* strains included in this study. Sensitive strains are marked with circles, *Sk-1* killers with stars and the one resistant strain with a square. Numbers in symbols represent sample-numbers at each site.

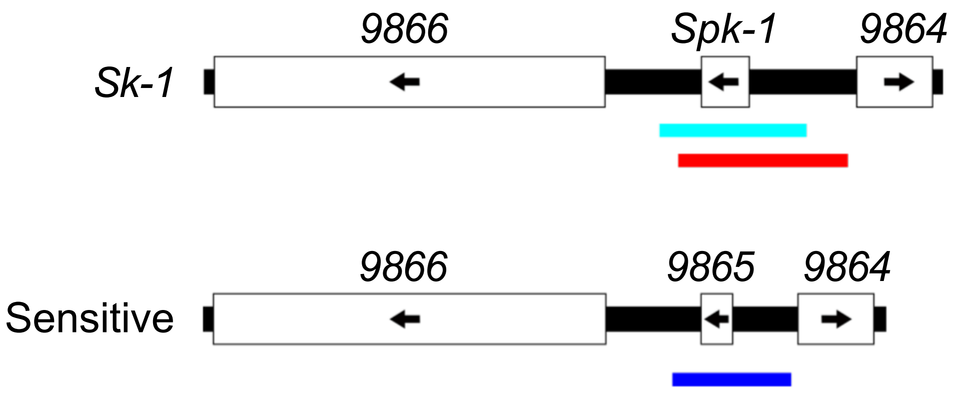

Figure S3: Deletion and insertion of the active element

The causative factor behind the *Sk-1 spore killing phenotype* is located between genes *NCU09866* and *NCU09864* on chromosome 6. A diagram of the *NCU09866-NCU09864* locus in *Sk-1* (top) strains and sensitive (bottom) strains is shown. The spore killing phenotype of an *Sk-1* strain can be eliminated by deleting a 2.8 kb interval (cyan) spanning the putative *Sk-1* gene. A spore killing phenotype can be established in a sensitive strain by replacing a 2.3 kb interval spanning *NCU0965* (blue) with the allelic 3.2 kb interval from an *Sk-1* strain (red).

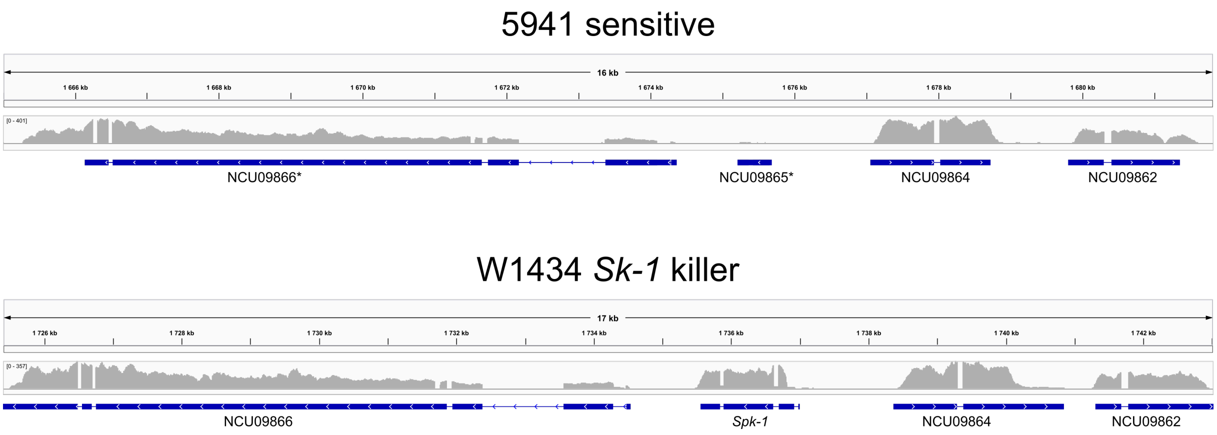

Figure S4: Deletion and insertion of the active element

Transcript levels at the *sk1c1* locus during vegetative growth. Inferred transcripts are generated using CUFFLINKS, and for genes marked with an asterisk (*), the inferred transcript structure is incomplete. *NCU09865* is not transcribed to a detectable level during either vegetative nor sexual growth in sensitive strain 5941, but *Spk-1* produces high levels of transcripts under both conditions.

A

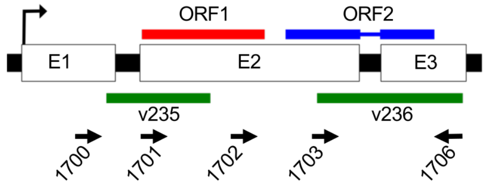

B

```
CGGACCTCCGAACCTACCTCTAGGTACATACGCAGTTGCAGAAGTGAGCTTCACTTGTACAGCAAGGGTAACTGGTCATAAG
GATGAAAGCAAACAGATAGGAACCTCAACCTGGGCCCCCTCAGCAGATATTTAAACTTCCCTATCCCCCCCTCTCAAATCCT
TCTCCCTCCCACCAAACAATATTCTTTCCACCAACTTCTCCCCACTCTCGTTTTGCAAGCAACATAATTGTACAAACATCGT
CAAGGCTCGAAAAGAAACCACTTGCGTCACCAAGAGTTTGACTTGAACCAAGCCCACCCGCCAGGTATGTTTCTTACTTACT
ATTCTACTTTCTATTTTCCTGTTTCTCCAACTCCTTTCTAACATGATAGTAAATGTCGCAGAATGAACAACACCACCTTGCC
CGTACCAGTCCCCATTTCGTGCGATGGCAACCCCAGCGTGCAATGGTATGTTTGGCTCATCGCAAGCATTGCATGGTGTGCA
GTGGGCTGCATATGGTTTTGGCATCGGTCCGAAACCGTTTGGACCTGGCTGAATGGTCCCATCGAGACATTCCACACCGAGC
AGCCGATGACCAGACGCTCGCGAATCACCACAGCCTGTTTCGTCTCAGTCTTGGTCGTTGCCCTGTGGCCATTGGAGATTTT
AATCTCCAACATTTGGTCAAGGGTCACGCACGCGACAAACAACCAAGGAACACCAAAAGACCAACCGAGAACGCAACCAGCA
GAAGAACCCCAAGCGGCACCCCCGCCGCCATATGATGTCGCAGTGGCCGCCGCATAGGCTCTTGGGGAGACGTGGTTGGTTT
CGACAGGAACCGTCTGGTTTGGAGGCTGGTGATACTGATCGATGAGCGAATTTTGGCACTACCTCGTCAACCTCGAGGCGGC
TCTCGGACAAGTCTTCAGGTCCCTTCGGCCGGGCGGCTTGTTTGTCTTTTCCGTCGAACACCCCATCTTCACGGCTCCTCAA
CGGCCGGGGTTCCAAAAGTCTGGAGCTGTCCAGGACTGGCCTTTGAGTAGTTACTTTGCCGAGGGAGAGCGGGAGGTCGACT
GGCTGGGTGCACCGGTGAGGAAGCAGCATCGAACCATTTCTAGGTCAGTAGCTCCGCGCTCCGAGCGAGGCTTGTGTGCTAA
CATGAGTTTCGTGCTCAAGCTATCTGAAGACCCTCCGTACAGTGGGCTTCGAAGTCGTTGTGGTGGACGAATGGGGTGTTAG
TGACGACGATAGCCGCAAACATCCCGACTGGCCAAATGATAGGGGGATTCCACGGTTTTTGCTCATCTCTGCGATAAAGAAA
GTCGATGGAGCGGATAGTCTGAAAACAACATTGGCTTGAGATTTGGTCTTGGTAACTGGATAAGTGGGCTCTAGTGTAACAG
GTAGACATGGTTTCCTCAGGTGGTTGATATTCGGCATGTAACTTTTCAGGCGAAGA
```

C

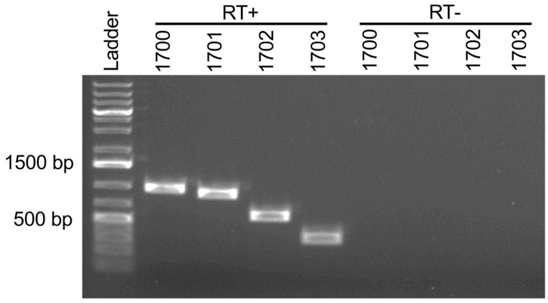

Figure S5: Dissection of the *Spk-1* transcript

(A) The *Spk-1* gene contains three exons (E1, E2, and E3) and two introns. The gene contains at least two potential open reading frames: ORF1 (red) and ORF2 (blue). The intervals deleted with vector 235 and vector 236 are indicated with green bars. The binding sites and directions of primers used in RT-PCR assays are indicated with arrows. **(B)** The *Spk-1* sequence is shown. Red and blue font are used for ORF1 and ORF2, respectively, and confirmed introns are highlighted in gray. The primer binding sites for primers 1700, 1701, 1702, 1703, and 1706 are underlined. **(C)** RT-PCR analysis was performed on total RNA from vegetative tissue of strain W1434. PCR was performed with the indicated forward primer (1700, 1701, 1702, 1703) and primer 1706 as the reverse primer. The image depicts products from each PCR reaction. Cloning and sequencing of the products in lanes "1700" and "1701" confirmed the locations of the two introns depicted in panels A and B (sequencing data is available at https://doi.org/10.6084/m9.figshare.14269781). No other introns were identified from the cloning and sequencing experiments. RT+, reverse transcriptase was used in the cDNA synthesis reaction; RT-, reverse transcriptase was left out of the cDNA synthesis reaction.

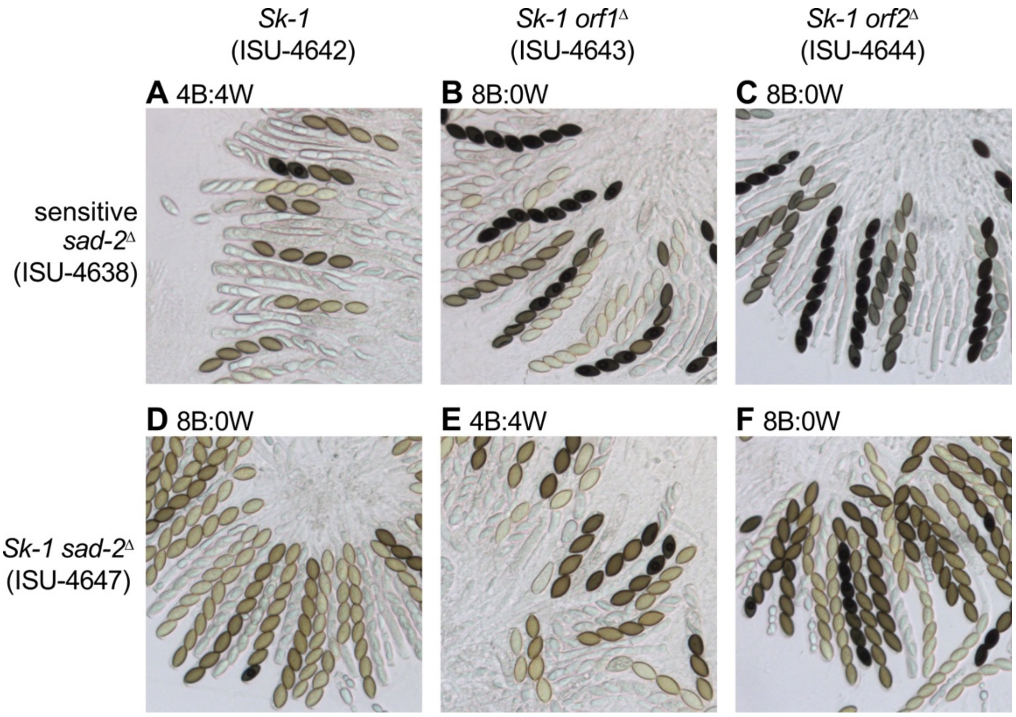

Figure S6: Ascus phenotypes of ORF deletions

Partial deletion of *Sk-1* ORF1 and *Sk-1* ORF2 have different effects on killing and resistance. The 5′ half of ORF1 was deleted with vector 235 to produce strain ISU-4643, and the 3′ 2/3 of ORF2 was deleted with vector 236 to produce strain ISU-4644. (A-F) Images are of asci from six crosses. The predominant phenotype is indicated above each image. The partial ORF1 deletion disrupted killing and resistance, while the partial ORF2 deletion disrupted killing but not resistance.

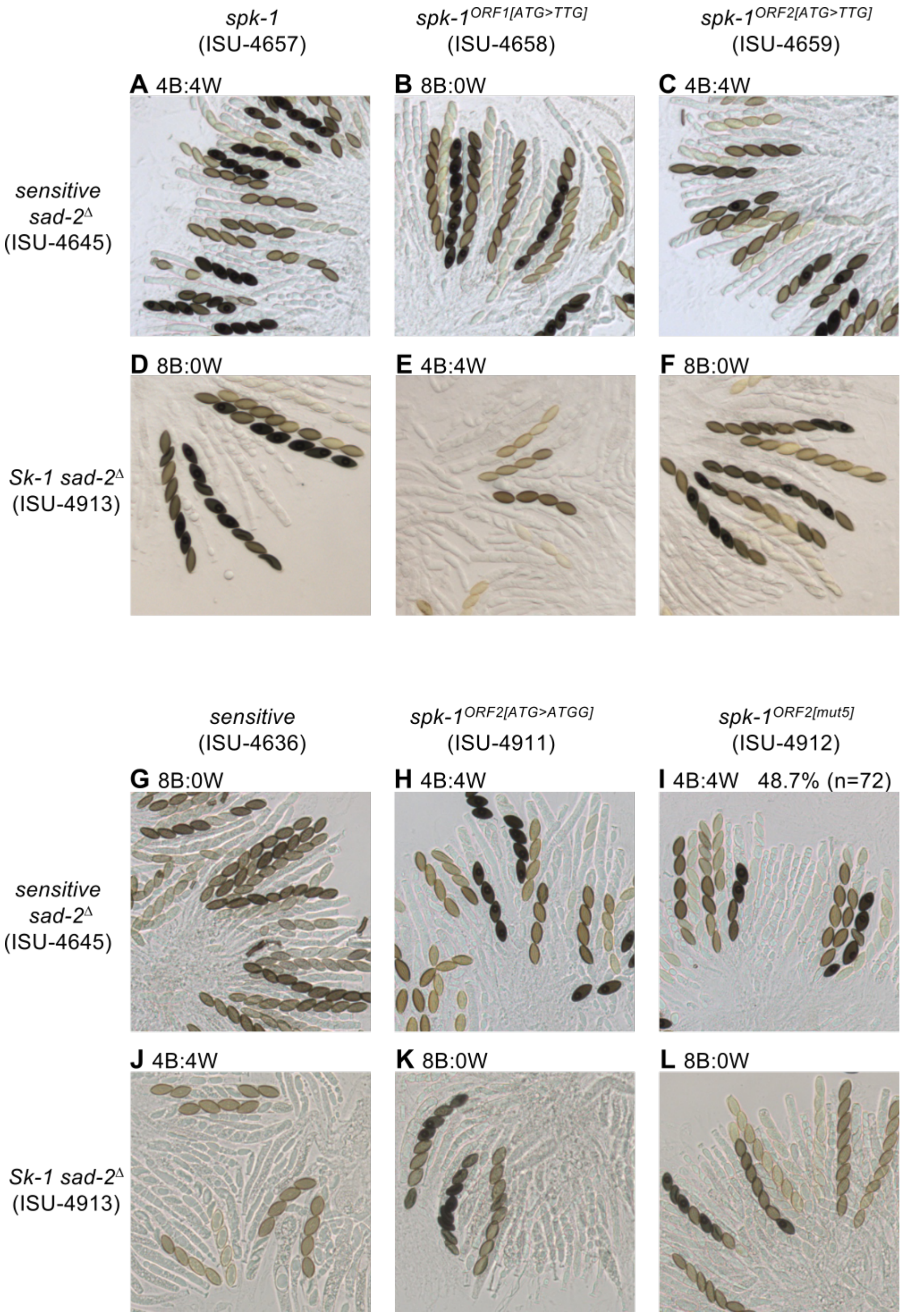

spk-1
(ISU-4657)
spk-1ORF1[ATG>TTG]
(ISU-4658)
spk-1ORF2[ATG>TTG]
(ISU-4659)
A 4B:4W
B 8B:0W
C 4B:4W
sensitive
sad-2Δ
(ISU-4645)
D 8B:0W
E 4B:4W
F 8B:0W
Sk-1 sad-2Δ
(ISU-4913)
sensitive
(ISU-4636)
spk-1ORF2[ATG>ATGG]
(ISU-4911)
spk-1ORF2[mut5]
(ISU-4912)
G 8B:0W
H 4B:4W
I 4B:4W 48.7% (n=72)
sensitive
sad-2Δ
(ISU-4645)
J 4B:4W
K 8B:0W
L 8B:0W
Sk-1 sad-2Δ
(ISU-4913)

M Location if insertions, deletions, and variants in *orf2[mut5]*.. *Intronic sequences are indicated with blue highlighting.*

```
              M  S  E  F  W  H  Y  L  V  N  L  E   A  A  L  G  Q  V  F  R
orf2          ATGAGCGAATTTTGGCACTACCTCGTCAACCTCGAG-GCGGCTCTCGGACAAGTCTTCAG  59
orf2[mut5]    ATGAGCGAATTTTGGCACTACCTCGTCAACCTCGAGTGAGGCTCTCGGACAAGTCTTCAG  60

               S  L  R   P  G  G  L  F  V  F  S  V  E  H  P  I  F  T  A
orf2          GTCCCTTCG-GCCGGGCGGCTTGTTTGTCTTTTCCGTCGAACACCCCATCTTCACGGCTC  118
orf2[mut5]    GTCCCTTCGTGCCGGGCGGCTTGTTTGTCTTT-CCGTCGAACACCCCATCTTCACG-CTC  118

              P  Q  R  P  G  F  Q  K   S  G  A  V  Q  D  W   P  L  S  S  Y
orf2          CTCAACGGCCGGGGTTCCAAAA-GTCTGGAGCTGTCCAGGACTG-GCCTTTGAGTAGTTA  176
orf2[mut5]    CTCAACGGCCGGGGTTCCAAATTGTCTGGAGCTGTCCAGGACTGTGCCTTTGAGTAGTTA  178

               F  A  E  G  E  R  E  V  D  W  L  G  A  P  V  R   K  Q  H
orf2          CTTTGCCGAGGGAGAGCGGGAGGTCGACTGGCTGGGTGCACCGGTGAGG-AAGCAGCATC  235
orf2[mut5]    CTTTGCC-AGGGAGAGCGGGAGGTCGAC-GGCTGGGTGCACCGGTGAGGTAAGCAGCATC  236

              R  T  I  S  S
orf2          GAACCATTTCTAGGTCAGTAGCTCCGCGCTCCGAGCGAGGCTTGTGTGCTAACATGAGTT  295
orf2[mut5]    GAACCATTTCTAGGTCAGTAGCTCCGCGCTCCGAGCGAGGCTTGTGTGCTAACATGAGTT  296

                          Y  L  K  T  L  R  T   V  G  F  E  V  V  V  V  D
orf2          TCGTGCTCAAGCTATCTGAAGACCCTCCGTAC-AGTGGGCTTCGAAGTCGTTGTGGTGGA  354
orf2[mut5]    TCGTGCTCAAGCTATCTGAAGACCCTCCGTACTAGTGGGCTTCGAAGTCGTTGTG-TGGA  355

               E  W  G  V  S  D  D  D  S  R  K  H  P  D  W  P  N  D   R
orf2          CGAATGGGGTGTTAGTGACGACGATAGCCGCAAACATCCCGACTGGCCAAATGATA-GGG  413
orf2[mut5]    CGAGTGGGGTGTTAGTGACGACGATAGCC-CAAACATCCCGACTGGCCAAGTGATAAGGG  414

              G  I  P  R  F  L  L   I  S  A  I  K  K  V  D  G  A  D  S  L
orf2          GGATTCCACGGTTTTTGCTC-ATCTCTGCGATAAAGAAAGTCGATGGAGCGGATAGTCTG  472
orf2[mut5]    GGATTCCACGGTTTTTGCTCTATCTCTGCGATAAA---AGTCGAAGG-GCGGATAGTCTG  470

               K  T  T  L   A  *
orf2          AAAACAACATT-GGCTTGA  490
orf2[mut5]    AAAACAACATTTGGCTTGA  489
```

Figure S7: Ascus phenotypes of mutating the start codons

Mutation of the *Spk-1* ORF1 start codon eliminates spore killing and resistance to spore killing. Strains ISU-4657, ISU-4658, and ISU-4659 were generated by inserting an *Spk-1* transgene or an *Spk-1* mutant transgene at the allelic location in a sensitive strain. **(A-F)** Images are of asci from six crosses. The predominant phenotype is indicated above each image. Mutation of the ORF1 start codon eliminated both killing and resistance, while mutation of the ORF2 start codon had no effect on killing or resistance. When introducing a frameshift mutation in ORF2 neither killing **(I)** nor resistance **(K)** is affected, but when generating a more complex ORF2 mutant (ORF2[mut5]) which introduced five single-nucleotide mutations, nine single-nucleotide insertions, seven single-nucleotide deletions, and a three-nucleotide long deletion **(M)**, we saw no effect on resistance but a reduced efficiency in killing. This reduced efficiency manifested itself as an excess in 5, 6 or 7-spored asci rather than a complete loss of killing ability.

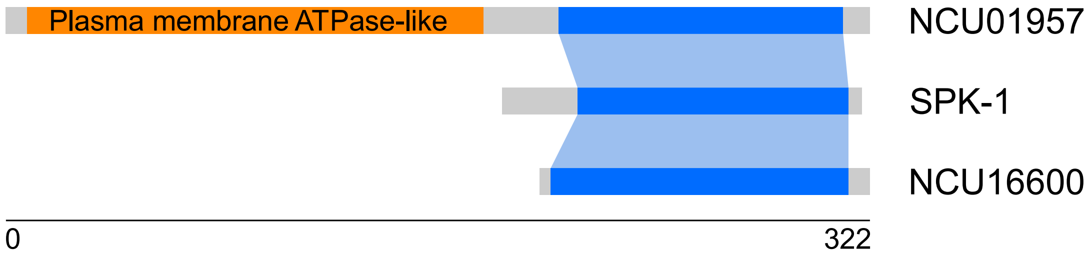

Figure S8: SPK-1 shows homology to NCU01957 and NCU16600

Cartoon of homologous regions of SPK-1 and the two annotated *N. crassa* genes NCU01957 and NCU16600. All three genes share the same homologous region which spans almost the entire sequence of SPK-1 and NCU16600, but only the C-terminal region of the longer NCU01957 gene. NCU01957 also contains a region with strong similarity to a *N. crassa* membrane ATPase gene, but this region is not found in the other two genes.

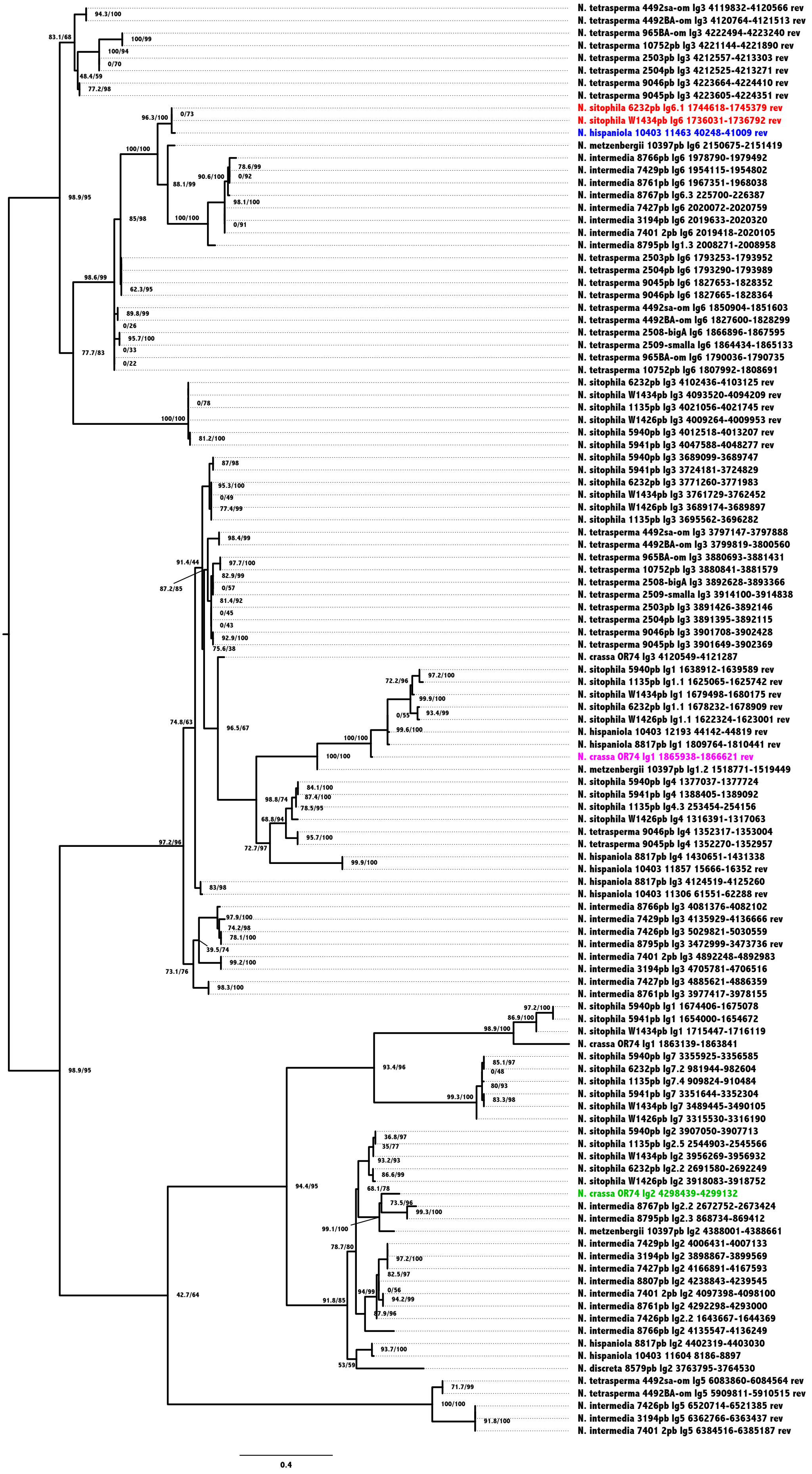
N. tetrasperma 4492sa-om lg3 4119832-4120566 rev
N. tetrasperma 4492BA-om lg3 4120764-4121513 rev
N. tetrasperma 965BA-om lg3 4222494-4223240 rev
N. tetrasperma 10752pb lg3 4221144-4221890 rev
N. tetrasperma 2503pb lg3 4212557-4213303 rev
N. tetrasperma 2504pb lg3 4212525-4213271 rev
N. tetrasperma 9046pb lg3 4223664-4224410 rev
N. tetrasperma 9045pb lg3 4223605-4224351 rev
N. sitophila 6232pb lg6.1 1744618-1745379 rev
N. sitophila W1434pb lg6 1736031-1736792 rev
N. hispaniola 10403 11463 40248-41009 rev
N. metzenbergii 10397pb lg6 2150675-2151419
N. intermedia 8766pb lg6 1978790-1979492
N. intermedia 7429pb lg6 1954115-1954802
N. intermedia 8761pb lg6 1967351-1968038
N. intermedia 8767pb lg6.3 225700-226387
N. intermedia 7427pb lg6 2020072-2020759
N. intermedia 3194pb lg6 2019633-2020320
N. intermedia 7401 2pb lg6 2019418-2020105
N. intermedia 8795pb lg1.3 2008271-2008958
N. tetrasperma 2503pb lg6 1793253-1793952
N. tetrasperma 2504pb lg6 1793290-1793989
N. tetrasperma 9045pb lg6 1827653-1828352
N. tetrasperma 9046pb lg6 1827665-1828364
N. tetrasperma 4492sa-om lg6 1850904-1851603
N. tetrasperma 4492BA-om lg6 1827600-1828299
N. tetrasperma 2508-bigA lg6 1866896-1867595
N. tetrasperma 2509-smalla lg6 1864434-1865133
N. tetrasperma 965BA-om lg6 1790036-1790735
N. tetrasperma 10752pb lg6 1807992-1808691
N. sitophila 6232pb lg3 4102436-4103125 rev
N. sitophila W1434pb lg3 4093520-4094209 rev
N. sitophila 1135pb lg3 4021056-4021745 rev
N. sitophila W1426pb lg3 4009264-4009953 rev
N. sitophila 5940pb lg3 4012518-4013207 rev
N. sitophila 5941pb lg3 4047588-4048277 rev
N. sitophila 5940pb lg3 3689099-3689747
N. sitophila 5941pb lg3 3724181-3724829
N. sitophila 6232pb lg3 3771260-3771983
N. sitophila W1434pb lg3 3761729-3762452
N. sitophila W1426pb lg3 3689174-3689897
N. sitophila 1135pb lg3 3695562-3696282
N. tetrasperma 4492sa-om lg3 3797147-3797888
N. tetrasperma 4492BA-om lg3 3799819-3800560
N. tetrasperma 965BA-om lg3 3880693-3881431
N. tetrasperma 10752pb lg3 3880841-3881579
N. tetrasperma 2508-bigA lg3 3892628-3893366
N. tetrasperma 2509-smalla lg3 3914100-3914838
N. tetrasperma 2503pb lg3 3891426-3892146
N. tetrasperma 2504pb lg3 3891395-3892115
N. tetrasperma 9046pb lg3 3901708-3902428
N. tetrasperma 9045pb lg3 3901649-3902369
N. crassa OR74 lg3 4120549-4121287
N. sitophila 5940pb lg1 1638912-1639589 rev
N. sitophila 1135pb lg1.1 1625065-1625742 rev
N. sitophila W1434pb lg1 1679498-1680175 rev
N. sitophila 6232pb lg1.1 1678232-1678909 rev
N. sitophila W1426pb lg1.1 1622324-1623001 rev
N. hispaniola 10403 12193 44142-44819 rev
N. hispaniola 8817pb lg1 1809764-1810441 rev
N. crassa OR74 lg1 1865938-1866621 rev
N. metzenbergii 10397pb lg1.2 1518771-1519449
N. sitophila 5940pb lg4 1377037-1377724
N. sitophila 5941pb lg4 1388405-1389092
N. sitophila 1135pb lg4.3 253454-254156
N. sitophila W1426pb lg4 1316391-1317063
N. tetrasperma 9046pb lg4 1352317-1353004
N. tetrasperma 9045pb lg4 1352270-1352957
N. hispaniola 8817pb lg4 1430651-1431338
N. hispaniola 10403 11857 15666-16352 rev
N. hispaniola 8817pb lg3 4124519-4125260
N. hispaniola 10403 11306 61551-62288 rev
N. intermedia 8766pb lg3 4081376-4082102
N. intermedia 7429pb lg3 4135929-4136666 rev
N. intermedia 7426pb lg3 5029821-5030559
N. intermedia 8795pb lg3 3472999-3473736 rev
N. intermedia 7401 2pb lg3 4892248-4892983
N. intermedia 3194pb lg3 4705781-4706516
N. intermedia 7427pb lg3 4885621-4886359
N. intermedia 8761pb lg3 3977417-3978155
N. sitophila 5940pb lg1 1674406-1675078
N. sitophila 5941pb lg1 1654000-1654672
N. sitophila W1434pb lg1 1715447-1716119
N. crassa OR74 lg1 1863139-1863841
N. sitophila 5940pb lg7 3355925-3356585
N. sitophila 6232pb lg7.2 981944-982604
N. sitophila 1135pb lg7.4 909824-910484
N. sitophila 5941pb lg7 3351644-3352304
N. sitophila W1434pb lg7 3489445-3490105
N. sitophila W1426pb lg7 3315530-3316190
N. sitophila 5940pb lg2 3907050-3907713
N. sitophila 1135pb lg2.5 2544903-2545566
N. sitophila W1434pb lg2 3956269-3956932
N. sitophila 6232pb lg2.2 2691580-2692249
N. sitophila W1426pb lg2 3918083-3918752
N. crassa OR74 lg2 4298439-4299132
N. intermedia 8767pb lg2.2 2672752-2673424
N. intermedia 8795pb lg2.3 868734-869412
N. metzenbergii 10397pb lg2 4388001-4388661
N. intermedia 7429pb lg2 4006431-4007133
N. intermedia 3194pb lg2 3898867-3899569
N. intermedia 7427pb lg2 4166891-4167593
N. intermedia 8807pb lg2 4238843-4239545
N. intermedia 7401 2pb lg2 4097398-4098100
N. intermedia 8761pb lg2 4292298-4293000
N. intermedia 7426pb lg2.2 1643667-1644369
N. intermedia 8766pb lg2 4135547-4136249
N. hispaniola 8817pb lg2 4402319-4403030
N. hispaniola 10403 11604 8186-8897
N. discreta 8579pb lg2 3763795-3764530
N. tetrasperma 4492sa-om lg5 6083860-6084564 rev
N. tetrasperma 4492BA-om lg5 5909811-5910515 rev
N. intermedia 7426pb lg5 6520714-6521385 rev
N. intermedia 3194pb lg5 6362766-6363437 rev
N. intermedia 7401 2pb lg5 6384516-6385187 rev
0.4

Figure S9: Phylogenetic tree of *Spk-1* homologs

Maximum likelihood phylogeny of SPK-1 and homologs in 31 *Neurospora* strains from 7 different species (*N. crassa*, *N. discreta*, *N. hispaniola*, *N. intermedia*, *N. metzenbergii*, *N. sitophila* and *N. tetrasperma*). SPK-1 from the two *N. sitophila Sk-1* strains are marked in red. *N. hispaniola* strain 10403 is the only other strain that carries a highly similar sequence (98.5% amino acid sequence identity, marked in blue), which is also found at the same locus on chromosome 6. Other homologous sequences range in amino acid sequence identity between 24% and 87%. Two annotated genes in the *N. crassa* OR74 reference genome are found among this set of sequences: *NCU01957* (also known as *AR2*, marked in magenta) and *NCU16600* (marked in green). *NCU01957* contains a Plasma Membrane ATPase domain not found in SPK-1 and is known to cause empty asci when mutated (Randall & Metzenberg 1998), but has otherwise no known function. *NCU16600* is also of unknown function.

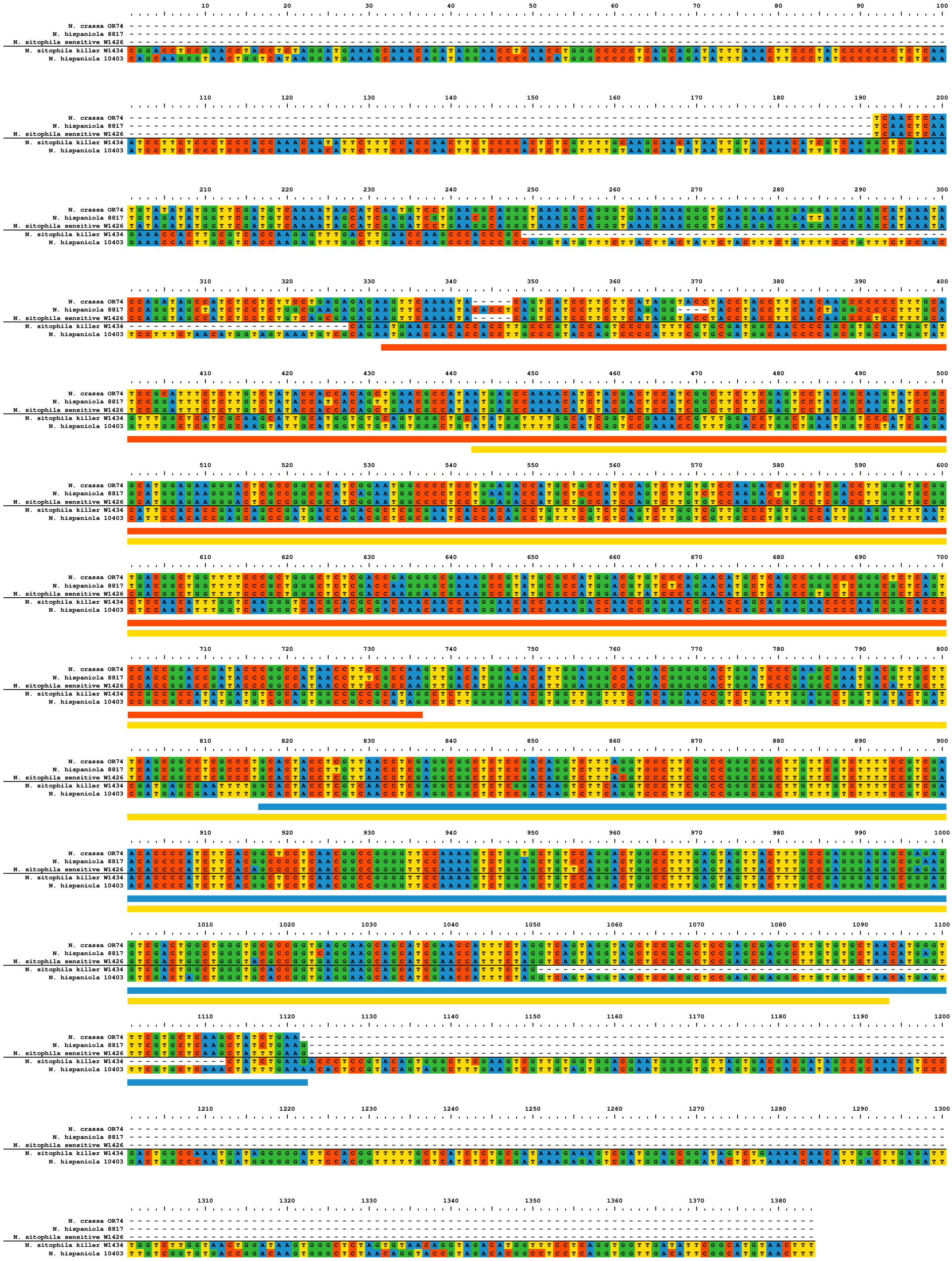

Figure S10: Alignment of *Spk-1* and *NCU09865* transcripts

Multiple sequence alignment of *NCU09865* from *N. crassa* OR74, *N. hispaniola* 8817 and *N. sitophila* W1426 (sensitive) and the *Spk-1* transcript from *N. sitophila* W1434 (killer) and *N.*

hispaniola 10403. Only the region marked with a blue block (position 917-1122) is actually aligning between *NCU09865* and *Spk-1*, and corresponds to the truncated part of *NCU09865* that remains at the *Spk-1* locus. The red block (position 332-736) marks the open reading frame of the *Spk-1* transcript. The yellow block (position 443-1093) marks the open reading frame of *NCU09865* in *N. crassa*.

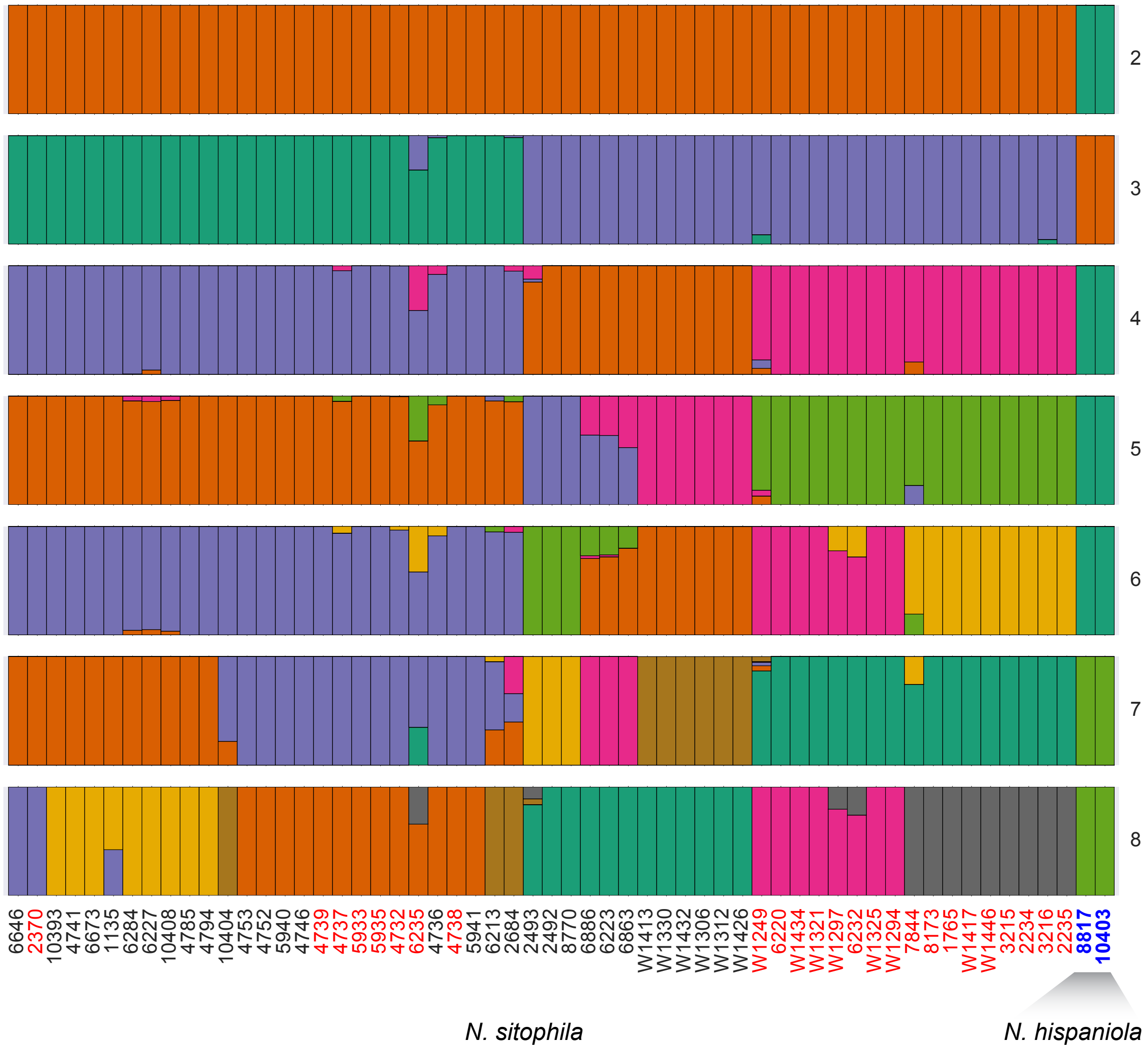

Figure S11: Admixture analysis of *N. sitophila* and *N. hispaniola*

An ADMIXTURE analysis of *N. sitophila* and *N. hispaniola* strains show no genome-wide signal of gene flow between the two species when varying number of clusters from 2 to 8. While *Spk-1* has been introgressed from *N. hispaniola*, geneflow must be rare.

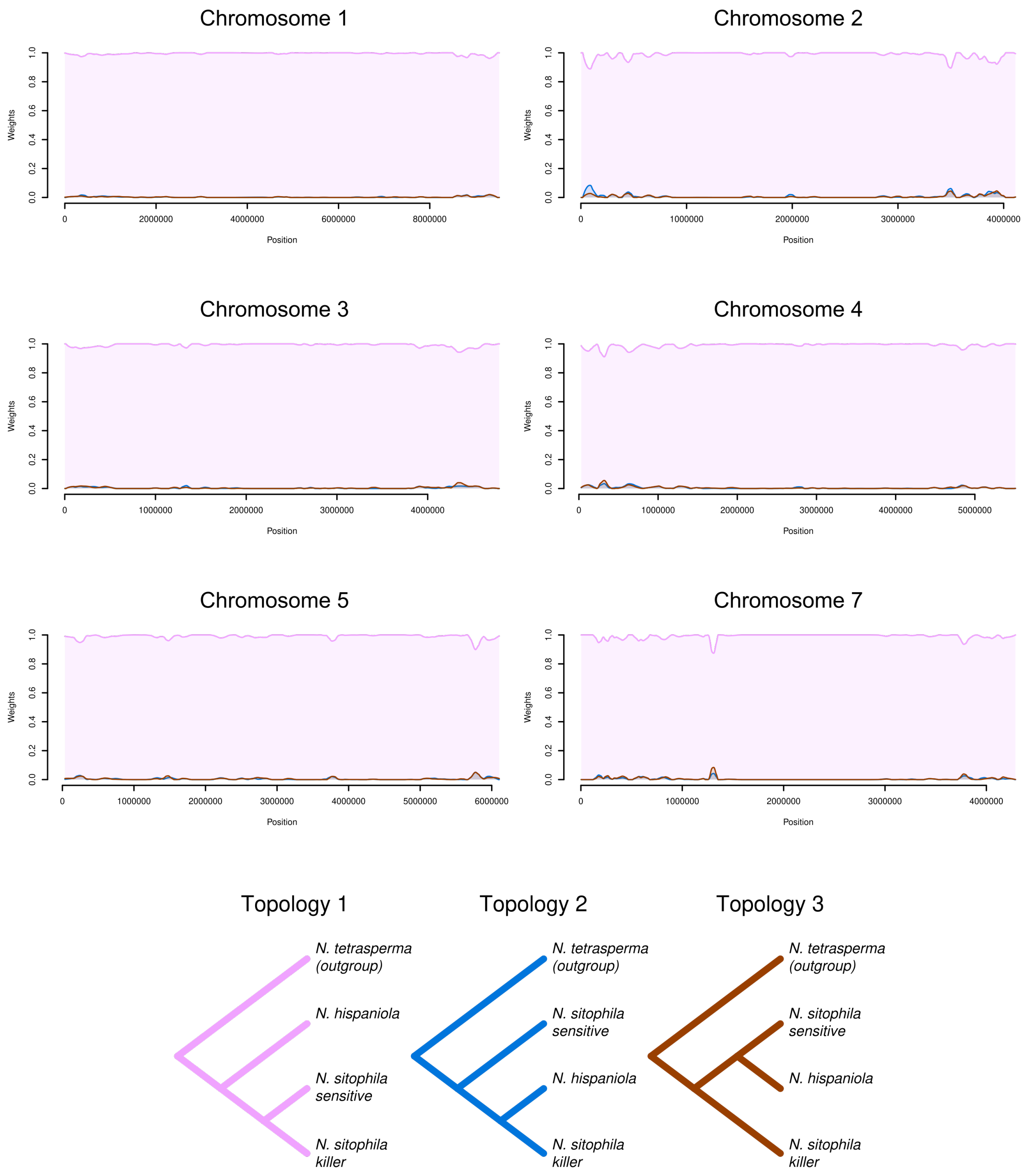

Figure S12: Local patterns of introgression across the genome

Twisst plots showing signals of local introgression from *N. hispaniola* across chromosome 1 to 5 and 7 (chromosome 6 is shown in Figure 2). The pink line shows the fraction of all tetrad trees that do not support introgression, while blue is consistent with introgression into *Sk-1* and brown with introgression into sensitive *N. sitophila* strains. All curves have been smoothed using Loess smoothing (span=0.05). Some small regions show a signal consistent with introgression, but no window has a stronger signal than *Spk-1*.

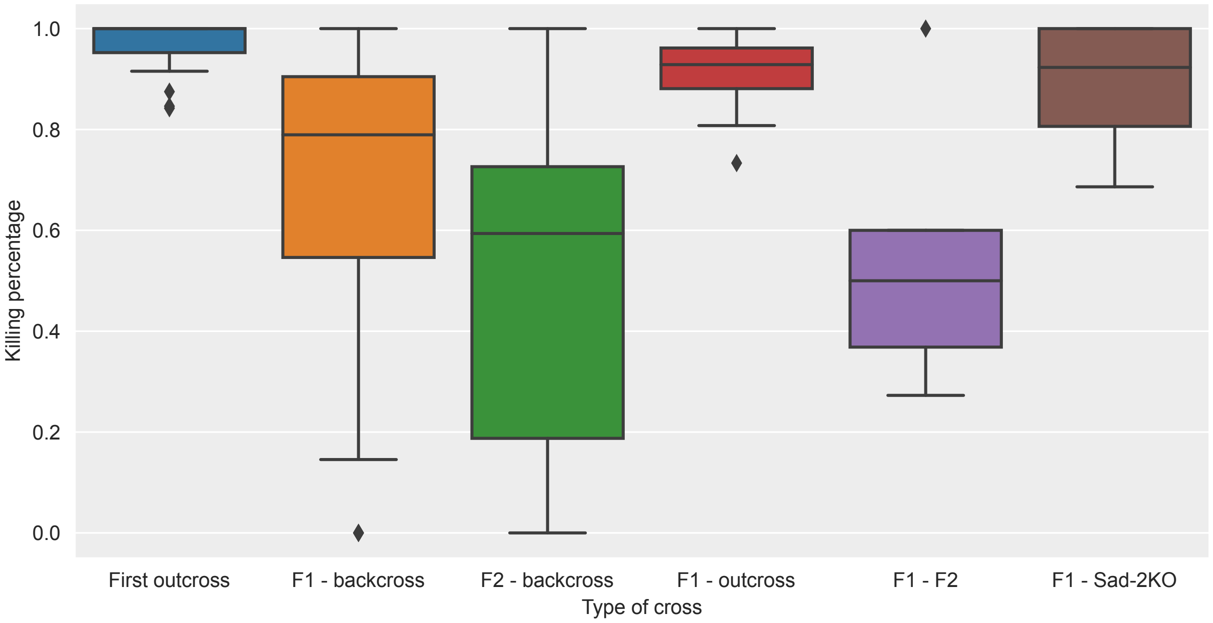

Figure S13: MSUD suppresses spore killing

Boxplot of fraction of asci containing 4 spores, indicating that spore killing has happened. Data is based on crosses between the sensitive Tahiti strains 4746 and 5940 and the killer strains 4738, 4739, W1325 and W1446. "Outcrosses" show the killing percentage in these crosses, "F1 - backcross" show the first backcross between the F1 and the sensitive parental strain and "F2 - backcross", the second backcross. "F1 - outcross" shows killing percentage of an outcross between a killer F1 with a nonparental sensitive strain. "F1 - F2" shows crosses between a killer F1 and a sensitive F2. "F1 - sad-2KO" shows the killing percentage in a cross between the killer F1 and its sensitive parent, where the *sad-2* gene has been deleted. This suppresses the MSUD system.

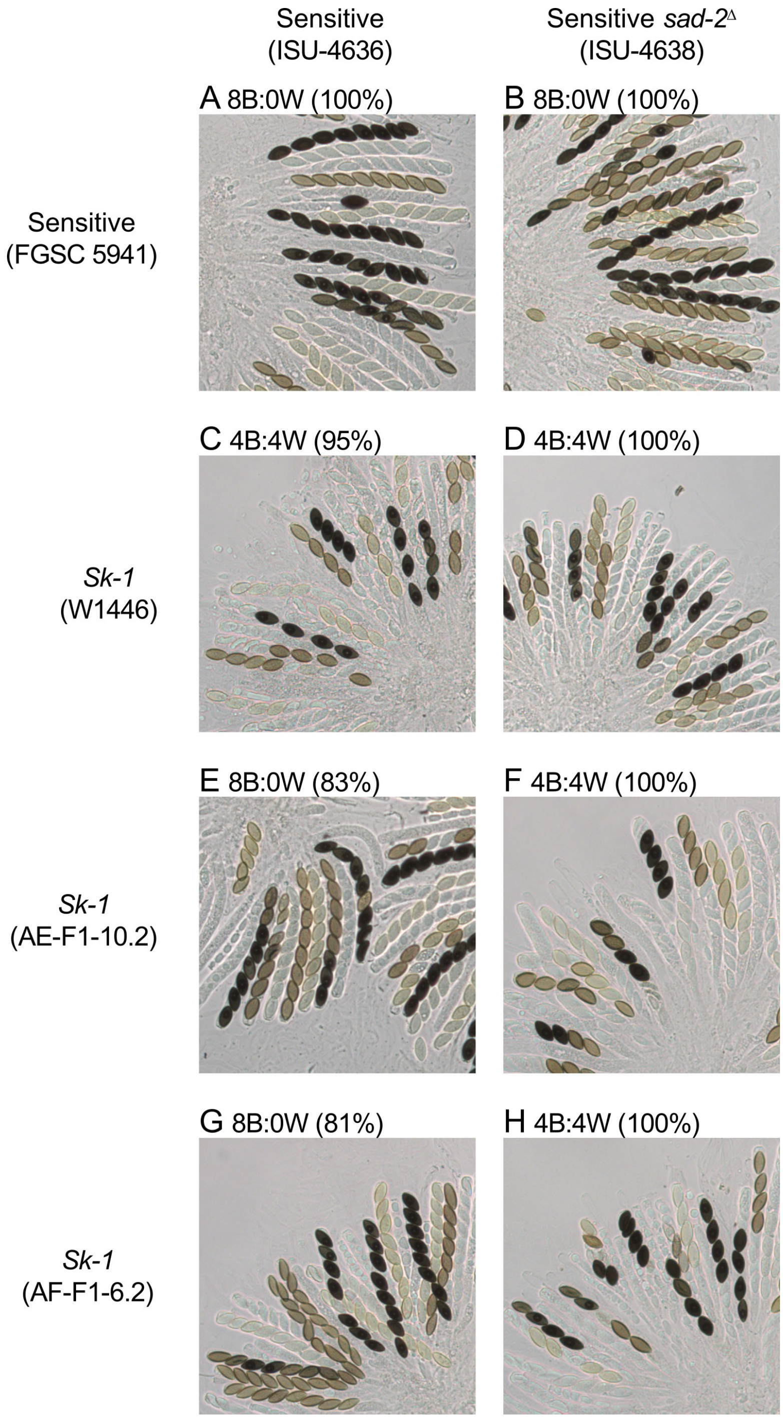

Figure S14 Ascus phenotype of MSUD suppressed spore killing

Suppression of MSUD increases the efficiency of spore killing in *Sk-1* × sensitive crosses. (A-H) The images are of rosettes from eight different crosses. The predominant ascus phenotype for each cross is listed above each image, along with the percentage of asci showing the predominant phenotype. AE-F1-10.2 is an F1 offspring from a cross between 4739 and 4746, and AF-F1-6.2 is an F1 offspring from a cross between 4738 and 4746.

Supplementary tables

Table S1: PacBio sequencing and genome assembly statistics for four *N. sitophila* strains

Strain [1]	Phenotype	Location	Mean coverage	Mean subread length (bp)	Assembly size (Mbp)	Contigs	Mapped contigs [2]
6232	*Sk-1*	Turkey	146	6038	39	27	15
1135	Sensitive	Panama	143	6175	41.2	42	20
W1426 [3]	*Sk-1*	Italy	93.5	7432	37.9	20	8
W1434 [3]	Sensitive	Italy	80.2	8197	39.2	14	7

[1] 1135 and 6232 are strain ID numbers in the Fungal Genetics Stock Center (FGSC). W1426 and W1434 are ID numbers from D. Jacobson's personal collection.

[2] Number of contigs mapping to the seven chromosomes of the *N. crassa* OR74A reference genome.

[3] Strain has been deposited to the Fungal Genetics Stock Center (FGSC). See Table S8 for FGSC accession number.

Table S2: Illumina HiSeq sequencing and assembly statistics

Species	Phenotype	Location	Strain	Contigs	Assembly size (bp)	N90	N80	N50	N20	Largest contig	Number of reads	Read length	Total data (Gbp)	Coverage
N. sitophila	*Sk-1*	Japan	1765	1953	38,707,971	15,821	25,744	55,111	114,458	371,488	8,612,848	124	2.14	55.2
N. sitophila	*Sk-1*	USA	2234	1863	38,485,632	17,629	30,042	71,689	135,644	272,731	10,913,177	124	2.71	70.3
N. sitophila	*Sk-1*	USA	2235	1805	38,772,771	17,922	29,059	64,944	121,534	319,446	12,202,102	124	3.03	78.0
N. sitophila	*Sk-1*	Hawaii	2370	1636	37,890,652	17,190	26,072	55,709	112,741	338,630	10,212,922	124	2.53	66.8
N. sitophila	Sensitive	India	2492	1904	38,128,085	17,125	27,456	62,599	121,274	448,962	12,570,109	124	3.12	81.8
N. sitophila	Sensitive	Malaya	2493	1747	38,881,544	18,233	28,089	66,516	122,833	351,117	11,389,221	124	2.82	72.6
N. sitophila	*Sk-1*	USA	2684	1371	38,159,651	21,773	33,360	74,598	145,207	950,644	10,429,030	124	2.59	67.8
N. sitophila	*Sk-1*	USA	3215	1730	38,297,183	18,010	28,739	69,997	140,229	295,300	9,679,280	124	2.40	62.7
N. sitophila	*Sk-1*	USA	3216	1828	38,623,104	17,025	28,503	60,409	119,478	304,077	9,465,706	124	2.35	60.8
N. sitophila	*Sk-1*	Tahiti	4732	1591	38,167,904	16,834	27,134	60,676	116,640	379,184	10,047,815	124	2.49	65.3
N. sitophila	Sensitive	Tahiti	4736	1906	38,155,269	18,038	28,819	63,253	125,960	396,913	14,128,440	124	3.50	91.8
N. sitophila	*Sk-1*	Tahiti	4737	1705	38,289,076	16,369	26,517	60,017	116,911	389,253	10,723,121	124	2.66	69.5
N. sitophila	*Sk-1*	Tahiti	4738	2081	37,961,582	16,009	26,042	56,517	114,157	379,162	11,263,599	124	2.79	73.6
N. sitophila	*Sk-1*	Tahiti	4739	1637	38,083,069	15,983	25,694	55,427	117,519	397,723	10,136,193	124	2.51	66.0
N. sitophila	Sensitive	Papua New Guinea	4741	2176	38,276,791	16,370	25,598	53,346	111,183	326,143	9,662,540	124	2.40	62.6
N. sitophila	Sensitive	Tahiti	4746	1915	37,979,133	15,506	25,547	55,239	114,037	255,821	9,632,767	124	2.39	62.9
N. sitophila	Sensitive	Tahiti	4752	2769	38,447,232	16,083	25,038	55,913	95,867	249,085	10,092,165	124	2.50	65.1
N. sitophila	Sensitive	Tahiti	4753	1700	37,981,914	17,728	27,775	60,433	118,535	414,591	10,960,501	124	2.72	71.6
N. sitophila	Sensitive	Haiti	4785	1622	37,896,451	16,375	23,869	53,142	103,698	396,448	10,313,457	124	2.56	67.5
N. sitophila	Sensitive	Haiti	4794	1462	37,917,033	18,260	28,253	64,044	122,270	414,635	10,455,431	124	2.59	68.4

Species	Phenotype	Location	Strain	Contigs	Assembly size (bp)	N90	N80	N50	N20	Largest contig	Number of reads	Read length	Total data (Gbp)	Coverage
N. sitophila	*Sk-1*	Tahiti	5933	2078	38,048,338	16,864	26,397	57,685	110,222	316,643	9,675,980	124	2.40	63.1
N. sitophila	*Sk-1*	Tahiti	5935	1923	38,151,115	16,972	26,217	55,129	115,663	389,311	9,566,368	124	2.37	62.2
N. sitophila	Sensitive	Tahiti	5940	2067	37,864,153	17,040	26,066	54,872	106,647	349,123	11,339,082	124	2.81	74.3
N. sitophila	Sensitive	Tahiti	5941	1701	37,867,531	17,078	27,426	60,198	130,267	262,542	10,771,936	124	2.67	70.5
N. sitophila	Sensitive	Rota	6213	1648	37,813,855	16,908	28,778	63,418	124,490	451,800	10,210,315	124	2.53	67.0
N. sitophila	*Sk-1*	USA	6220	1710	39,377,771	17,989	29,191	65,081	130,637	365,571	11,578,623	124	2.87	72.9
N. sitophila	Sensitive	Gabon	6223	1951	37,725,619	17,358	28,384	58,805	108,770	282,389	9,537,071	124	2.37	62.7
N. sitophila	Sensitive	Gabon	6227	3019	38,139,207	13,525	21,277	45,411	85,486	375,307	7,681,545	124	1.91	49.9
N. sitophila	*Sk-1*	Turkey	6232	2106	38,898,329	16,791	26,063	61,467	123,557	464,739	9,737,681	124	2.41	62.1
N. sitophila	*Sk-1*	Roratonga	6235	2070	38,313,666	15,580	25,215	56,854	115,919	282,713	9,119,530	124	2.26	59.0
N. sitophila	Sensitive	Ivory coast	6284	1549	37,850,405	17,836	28,310	60,190	121,625	471,273	9,509,623	124	2.36	62.3
N. sitophila	Sensitive	Mexico	6646	1548	37,917,104	17,406	27,952	61,709	113,577	413,832	9,655,422	124	2.39	63.2
N. sitophila	Sensitive	Brazil	6673	1618	37,932,419	16,753	26,308	54,172	108,750	262,918	8,904,810	124	2.21	58.2
N. sitophila	Sensitive	Hawaii	6689	10327	43,985,540	11,677	24,931	62,163	121,934	383,175	9,312,497	124	2.31	52.5
N. sitophila	Resistant	Gabon	6850	1629	39,107,064	16,857	28,995	60,589	111,166	290,727	9,339,131	124	2.32	59.2
N. sitophila	Sensitive	Ivory coast	6863	1645	37,789,059	17,514	28,023	59,451	114,104	283,786	9,865,808	124	2.45	64.7
N. sitophila	Sensitive	Gabon	6886	1892	37,863,691	16,625	26,786	60,446	114,693	378,496	9,039,032	124	2.24	59.2
N. sitophila	*Sk-1*	Australia	7844	1884	38,762,514	16,500	26,795	57,191	107,482	409,024	9,172,136	124	2.27	58.7
N. sitophila	*Sk-1*	Australia	8173	1834	38,493,069	17,297	26,291	59,376	119,284	279,482	9,896,780	124	2.45	63.8
N. sitophila	Sensitive	India	8770	1865	38,113,580	16,423	25,764	56,769	112,688	319,637	9,972,863	124	2.47	64.9
N. sitophila	Sensitive	Papua New Guinea	10393	1491	37,800,550	18,002	26,831	56,392	112,475	395,346	9,255,830	124	2.30	60.7

Species	Phenotype	Location	Strain	Contigs	Assembly size (bp)	N90	N80	N50	N20	Largest contig	Number of reads	Read length	Total data (Gbp)	Coverage
N. sitophila	Sensitive	Haiti	10404	1813	37,873,810	16,452	26,738	54,719	102,744	233,791	9,910,163	124	2.46	64.9
N. sitophila	Sensitive	Gabon	10408	1887	38,062,587	18,152	30,476	66,469	112,349	471,331	11,287,709	124	2.80	73.5
N. sitophila	*Sk-1*	Portugal	W1249 [1]	1465	39,251,111	16,537	26,375	57,314	110,291	363,664	9,533,549	124	2.36	60.2
N. sitophila	*Sk-1*	Spain	W1294 [1]	2329	39,072,114	15,077	23,893	51,772	102,729	494,286	10,188,425	124	2.53	64.7
N. sitophila	*Sk-1*	Switzerland	W1297 [1]	1930	39,216,768	16,185	25,216	56,776	107,778	554,470	9,694,420	124	2.40	61.3
N. sitophila	Sensitive	Italy	W1306 [1]	1828	37,708,235	16,451	26,515	57,583	99,977	296,869	9,734,942	124	2.41	64.0
N. sitophila	Sensitive	Italy	W1312 [1]	1821	37,699,980	16,753	27,118	61,471	113,498	425,044	10,853,639	124	2.69	71.4
N. sitophila	*Sk-1*	Italy	W1321 [1]	2069	39,410,520	15,969	25,193	58,514	110,590	420,290	9,682,315	124	2.40	60.9
N. crassa[2]	Unknown	Unknown	W1322 [1]	1883	37,188,009	17,387	28,626	59,733	116,387	306,637	9,978,456	124	2.47	66.5
N. sitophila	*Sk-1*	Italy	W1325 [1]	2175	39,108,556	16,752	26,205	57,541	112,927	321,839	8,882,548	124	2.20	56.3
N. sitophila	Sensitive	Italy	W1330 [1]	1827	37,733,606	15,518	25,334	57,073	99,718	424,769	10,153,224	124	2.52	66.7
N. sitophila	Sensitive	Italy	W1413 [1]	2114	37,600,533	12,686	20,141	41,628	87,404	293,508	9,301,378	124	2.31	61.3
N. sitophila	*Sk-1*	Italy	W1417 [1]	1805	38,625,776	16,389	26,201	60,780	116,147	381,804	11,101,624	124	2.75	71.3
N. sitophila	Sensitive	Italy	W1426 [1]	2018	37,649,517	13,626	21,600	45,376	88,860	293,454	7,766,246	124	1.93	51.2
N. sitophila	Sensitive	Italy	W1432 [1]	2040	37,712,739	13,481	22,012	45,587	90,778	293,510	7,537,534	124	1.87	49.6
N. sitophila	*Sk-1*	Italy	W1434 [1]	2258	38,831,428	12,628	20,340	41,441	88,905	300,902	7,398,586	124	1.83	47.3
N. sitophila	*Sk-1*	Italy	W1446 [1]	2036	38,623,459	14,092	22,116	51,420	104,107	598,164	7,793,787	124	1.93	50.0
N. hispaniola	Unknown	Haiti	10403	2154	41,172,793	22,332	34,629	71,246	132,013	433,467	10,136,566	124	2.51	61.1
N. perkinsii	Sensitive	Congo	8842	2266	42,659,328	20,719	35,046	74,666	147,897	327,068	9,702,211	124	2.41	56.4
N. perkinsii	Unknown	Gabon	10406	2089	43,673,595	21,438	35,654	74,160	140,814	427,677	10,105,447	124	2.51	57.4

[1] Strain has been deposited to the Fungal Genetics Stock Center (FGSC). See Table S8 for FGSC accession number.
[2] Strain W1322 was annotated as *N. sitophila*, but the sequenced strain clusters with *N. crassa* in phylogenetic analyses (potentially due to contamination), and it was therefore excluded from all further analyses.

Table S3: Ascus phenotype count data

	ISU-4645 × ISU-4636	ISU-4645 × ISU-4911	ISU-4645 × ISU-4912
	Sk^S sad-2^{Δ} × *Sk^S*	*Sk^S sad-2^{Δ}* × *Spk-$1^{ORF2[ATG>ATGG]}$*	*Sk^S sad-2^{Δ}* × *Spk-$1^{ORF2[mut5]}$*
4B:4W + 3B:5W	2	55	35
6B:2W + 5B:3W	1	0	32
8B:0W + 7B:1W	49	0	5

Intact asci with mature ascospores were examined from images of rosettes of the indicated crosses. The total number of asci of each phenotype are indicated.

Table S4: Phenotype summary

Phenotype	*spk-1*	*orf1$^{\Delta}$*	*orf1 ATG>TTG*	*orf2$^{\Delta}$*	*orf2 ATG>TTG*	*orf2 ATG>ATGG*	*orf2 mut5*
Killer	+	−	−	−	+	+	+/−
Resistant	+	−	−	+	+	+	+

killer (+) The predominant ascus phenotype in crosses of the indicated genotype with sensitive is 4B:4W. resistant (+) The predominant ascus phenotype in crosses of the indicated genotype with *Sk-1* is 8B:0W.

Table S5: *Spk-1* homologs in *Neurospora*

Species	Strain	Number of homologs [1]	Genomic location [2]
N. crassa	OR74 [3]	4	lg1 [1863139-1863841]<br>lg1 [1865938-1866621] rev<br>lg2 [4298439-4299132]<br>lg3 [4120549-4121287]
N. discreta	8579 [4]	1	lg2 [3763795-3764530]
N. hispaniola	10403 [5]	5	11306 [61551-62288] rev<br>11463 [40248-41009] rev<br>11604 [8186-8897]<br>11857 [15666-16352] rev<br>12193 [44142-44819] rev
N. hispaniola	8817 [4]	4	lg1 [1809764-1810441] rev<br>lg2 [4402319-4403030]<br>lg3 [4124519-4125260]<br>lg4 [1430651-1431338]
N. intermedia	3194 [6]	4	lg2 [3898867-3899569]<br>lg3 [4705781-4706516]<br>lg5 [6362766-6363437] rev<br>lg6 [2019633-2020320]
N. intermedia	7401 [6]	4	lg2 [4097398-4098100]<br>lg3 [4892248-4892983]<br>lg5 [6384516-6385187] rev<br>lg6 [2019418-2020105]
N. intermedia	7426 [6]	3	lg2.2 [1643667-1644369]<br>lg3 [5029821-5030559]<br>lg5 [6520714-6521385] rev
N. intermedia	7427 [6]	3	lg2 [4166891-4167593]<br>lg3 [4885621-4886359]<br>lg6 [2020072-2020759]
N. intermedia	7429 [6]	3	lg2 [4006431-4007133]<br>lg3 [4135929-4136666] rev<br>lg6 [1954115-1954802]
N. intermedia	8761 [6]	3	lg2 [4292298-4293000]<br>lg3 [3977417-3978155]<br>lg6 [1967351-1968038]
N. intermedia	8766 [6]	3	lg2 [4135547-4136249]<br>lg3 [4081376-4082102]<br>lg6 [1978790-1979492]
N. intermedia	8767 [6]	2	lg2.2 [2672752-2673424]<br>lg6.3 [225700-226387]
N. intermedia	8795 [6]	3	lg1.3 [2008271-2008958]<br>lg2.3 [868734-869412]<br>lg3 [3472999-3473736] rev

N. intermedia	8807<br>[7]	1	lg2 [4238843-4239545]
N. metzenbergii	10397<br>[4]	3	lg1.2 [1518771-1519449]<br>lg2 [4388001-4388661]<br>lg6 [2150675-2151419]
N. sitophila	1135<br>[8]	6	lg1.1 [1625065-1625742] rev<br>lg2.5 [2544903-2545566]<br>lg3 [3695562-3696282]<br>lg3 [4021056-4021745] rev<br>lg4.3 [253454-254156]<br>lg7.4 [909824-910484]
N. sitophila	5940<br>[9]	6	lg1 [1638912-1639589] rev<br>lg1 [1674406-1675078]<br>lg2 [3907050-3907713]<br>lg3 [3689099-3689747]<br>lg3 [4012518-4013207] rev<br>lg4 [1377037-1377724]<br>lg7 [3355925-3356585]
N. sitophila	5941<br>[9]	5	lg1 [1654000-1654672]<br>lg3 [3724181-3724829]<br>lg3 [4047588-4048277] rev<br>lg4 [1388405-1389092]<br>lg7 [3351644-3352304]
N. sitophila	6232<br>[8]	6	lg1.1 [1678232-1678909] rev<br>lg2.2 [2691580-2692249]<br>lg3 [3771260-3771983]<br>lg3 [4102436-4103125] rev<br>lg6.1 [1744618-1745379] rev<br>lg7.2 [981944-982604]
N. sitophila	W1426<br>[8]	6	lg1.1 [1622324-1623001] rev<br>lg2 [3918083-3918752]<br>lg3 [3689174-3689897]<br>lg3 [4009264-4009953] rev<br>lg4 [1316391-1317063]<br>lg7 [3315530-3316190]
N. sitophila	W1434<br>[8]	7	lg1 [1679498-1680175] rev<br>lg1 [1715447-1716119]<br>lg2 [3956269-3956932]<br>lg3 [3761729-3762452]<br>lg3 [4093520-4094209] rev<br>lg6 [1736031-1736792] rev<br>lg7 [3489445-3490105]
N. tetrasperma	2508<br>[3]	2	lg3 [3892628-3893366]<br>lg6 [1866896-1867595]
N. tetrasperma	2509<br>[3]	2	lg3 [3914100-3914838]<br>lg6 [1864434-1865133]

N. tetrasperma	4492A / 9033 [7]	4	lg3 [3799819-3800560]<br>lg3 [4120764-4121513] rev<br>lg5 [5909811-5910515] rev<br>lg6 [1827600-1828299]
N. tetrasperma	4492a / 9034 [7]	4	lg3 [3797147-3797888]<br>lg3 [4119832-4120566] rev<br>lg5 [6083860-6084564] rev<br>lg6 [1850904-1851603]
N. tetrasperma	965A [7]	3	lg3 [3880693-3881431]<br>lg3 [4222494-4223240] rev<br>lg6 [1790036-1790735]
N. tetrasperma	10752 [7]	3	lg3 [3880841-3881579]<br>lg3 [4221144-4221890] rev<br>lg6 [1807992-1808691]
N. tetrasperma	2503 [7]	3	lg3[3891426-3892146]<br>lg3 [4212557-4213303] rev<br>lg6 [1793253-1793952]
N. tetrasperma	2504 [7]	3	lg3 [3891395-3892115]<br>lg3 [4212525-4213271] rev<br>lg6 [1793290-1793989]
N. tetrasperma	9045 [7]	4	lg3 [3901649-3902369]<br>lg3 [4223605-4224351] rev<br>lg4 [1352270-1352957]<br>lg6 [1827653-1828352]
N. tetrasperma	9046 [7]	4	lg3 [3901708-3902428]<br>lg3 [4223664-4224410] rev<br>lg4 [1352317-1353004]<br>lg6 [1827665-1828364]

[1] Number of hits longer than 100 aa, when searching genome assembly using tblastn.
[2] Location of all tblastn hits. Contigs are named after linkage group / chromosome (for instance lg3 means linkage group 3) except in the case of strain 10403, where the contigs have not been placed on a linkage group. Coordinates for the blastn hits at listed within brackets. Hits found in the reverse strand are marked “rev”.
[3] Genome assembly available at https://fungidb.org/
[4] Genome assembly available at https://doi.org/10.6084/m9.figshare.c.4310996
[5] Genome assembly available at https://doi.org/10.6084/m9.figshare.12820754
[6] Genome assembly available at https://doi.org/10.6084/m9.figshare.6974033.v1
[7] Genome assembly available at https://doi.org/10.6084/m9.figshare.5327515.v1
[8] Genome assembly available at https://doi.org/10.6084/m9.figshare.12820811
[9] Genome assembly available at https://doi.org/10.6084/m9.figshare.7346591.v1

Table S6: Tester strains for phenotyping spore killing in natural *N. sitophila* isolates

FGSC number [1]	Mating type	Phenotype
4762	A	Killer
4763	a	Killer
4887	A	Sensitive
4888	a	Sensitive

All tester strains carry the *fluffy* mutation, which increases fertility and reduces conidiation.
[1] Strain ID numbers at the Fungal Genetics Stock Center.

Table S7: *N. sitophila* tester strains

Name (alias)	Genotype
AF-F1-6.2	*Sk-1 a* (Recombinant from FGSC 4739 × FGSC 4746)
AE-F1-10.2	*Sk-1 a* (Recombinant from FGSC 4738 × FGSC 4746)
FGSC 4738	*Sk-1 a* (Tahiti, 1983)
FGSC 4739	*Sk-1 a* (Tahiti, 1983)
FGSC 4746	*A* (Tahiti)
FGSC 5941	*a* (Tahiti, 1983)
FGSC 26716 [1]	*mus-51*$^{\Delta}$*::nat1 A* (Deletion of *mus-51* from FGSC 4746)
FGSC 26717 [1]	*mus-51*$^{\Delta}$*::nat1 A* (Deletion of *mus-51* from W1426)
FGSC 26758 / W1426	*A* (Italy)
FGSC 26760 / W1434	*Sk-1 A* (Italy)
FGSC 26761 / W1446	*Sk-1 a* (Italy)
ISU-4296 (HDS45.4.2)	*Sk-1*$^{\Delta}$*::hph A* (Transformation of W1434 with vector 134)
ISU-4529 (HNR143.2.1)	*mus-51*$^{\Delta}$*::nat1; ncu09865*$^{\Delta}$::*Sk-1-hph A* (Transformation of ISU-4637 with vector 205a)
ISU-4638 (HNR119.1.1)	*mus-51*$^{\Delta}$*::nat1; sad-2*$^{\Delta}$*::hph; A* (Transformation of ISU-4636 with vector 178).
ISU-4641 (RNR167.28)	*mus-51*$^{\Delta}$*::nat1 a* (Recombinant from ISU-4638 × FGSC 5941)
ISU-4642 (RNR173.5)	*mus-51*$^{\Delta}$*::nat1; Sk-1 a* (Recombinant from W1434 × ISU-4641)
ISU-4643 (HNR176.1.1)	*mus-51*$^{\Delta}$*::nat1; Sk-1 orf1*$^{\Delta}$*::hph a* (Transformation of ISU-4642 with v235)
ISU-4644 (HNR177.1.1)	*mus-51*$^{\Delta}$*::nat1; Sk-1 orf2*$^{\Delta}$*::hph a* (Transformation of ISU-4642 with v236)
ISU-4645 (HNR167.17)	*mus-51*$^{\Delta}$*::nat1; sad-2*$^{\Delta}$*::hph a* (Recombinant from ISU-4638 × FGSC 5941)
ISU-4646 (RNR173.2)	*mus-51*$^{\Delta}$*::nat1; Sk-1 A* (Recombinant from W1434 × ISU-4641)
ISU-4647 (RNR226.4)	*mus-51*$^{\Delta}$*::nat1; sad-2*$^{\Delta}$*::hph; Sk-1 A* (Recombinant from ISU-4638 × W1434)
ISU-4657 (HNR222.1.1)	*mus-51*$^{\Delta}$*::nat1; ncu09865*$^{\Delta}$::*Sk-1-hph A* (Transformation of ISU-4636 with v205a)
ISU-4658 (HNR223.1.1)	*mus-51*$^{\Delta}$*::nat1; ncu09865*$^{\Delta}$::*Sk-1*$^{ORF1[ATG>TTG]}$*-hph A* (Transformation of ISU-4636 with v205b)
ISU-4659 (HNR224.3.2)	*mus-51*$^{\Delta}$*::nat1; ncu09865*$^{\Delta}$::*Sk-1*$^{ORF2[ATG>TTG]}$*-hph A* (Transformation of ISU-4636 with v205c)
ISU-4911 (CNR 305.3.1)	*mus-51*$^{\Delta}$*::nat1; ncu09865*$^{\Delta}$::*spk-1*$^{ORF2[ATG>ATGG]}$*-hph A* (Transformation of ISU-4636 with v205d)
ISU-4912 (CNR306.2.1)	*mus-51*$^{\Delta}$*::nat1; ncu09865*$^{\Delta}$::*spk-1*$^{ORF2\ [mut5]}$*-hph A* (Transformation of ISU-4636 with v205e)

ISU-4913 (RNR226.2)	*mus-51*$^{\Delta}$*::nat1; sad-2*$^{\Delta}$*::hph; Sk-1 a* (Recombinant from ISU-4638 × W1434)

[1] Rhoades, N. A., E.K. Webber, and T.M. Hammond (2020) "A Nonhomologous End-Joining Mutant for Neurospora sitophila Research," *Fungal Genetics Reports*: Vol. 64, Article 1. https://doi.org/10.4148/1941-4765.2172

Table S8: Strains deposited to FGSC [1]

Strain	FGSC accession number
W1249	26747
W1294	26748
W1297	26749
W1306	26750
W1312	26751
W1321	26752
W1322	26753
W1325	26754
W1330	26755
W1413	26756
W1417	26757
W1426	26758
W1432	26759
W1434	26760
W1446	26761
ISU-4646/RNR 173.2	26764
ISU-4642/RNR 173.5	26765
ISU-4647/RNR 226.4	26798
ISU-4648/RNR 226.2	26797
ISU-4645/RNR 167.17	26796
ISU-4638/HNR 119.1.1	26795

[1] Fungal Genetics Stock Center. http://fgsc.net/

Table S9: Vector construction details

Primer Number	Sequence (5' > 3')	
v134 (hph)	to delete a 2.8 kbp DNA interval spanning sk1c1[k]	
12	AACTGATATTGAAGGAGCATTTTTTGG	C
13	AACTGGTTCCCGGTCGGCAT	C
1205	GCCGGGATGGGATACAAGACG	L
1206	AAAAAATGCTCCTTCAATATCAGTTCCAACCTTCTGCGCGACCAT	L
1207	GAGTAGATGCCGACCGGGAACCAGTTTTGTCTCAGCCGTTCCGCTCCT	R
1208	TTGAAAGATGGGTGGGTGAAAAGA	R
1209	GAGCGGCAACAGTAATCGTAGTGG	N
1210	CGTGGGCAAGTCAAGTCCCTCA	N
v178 (hph)	to delete *sad-2* coding region	
12	AACTGATATTGAAGGAGCATTTTTTGG	C
13	AACTGGTTCCCGGTCGGCAT	C
1465	CCTTTAACTCCTCTCCACTCGCTTG	L
1466	AAAAAATGCTCCTTCAATATCAGTTACTAGGGCCGCCTGGACTGGAT	L
1467	GAGTAGATGCCGACCGGGAACCAGTTCGAAGCAGTGTAGAAGGGGGAAGA	R
1468	CACCAACCACCACCACTTCGAC	R
1469	TCATCCCTTGTTGTTCGCGATG	N
1470	ACCTCCCAACCAACGCCTTCTC	N
v205a (Spk-1-hph),<br>*v205b (Spk-1$^{ORF1[ATG>TTG]}$-hph)*<br>*v205c (Spk-1$^{ORF2[ATG>TTG]}$-hph)*<br>*v205d (Spk-1$^{ORF2[ATG>ATGG]}$-hph)*<br>*v205e (Spk-1$^{ORF2[mut5]}$-hph)*	to insert *Spk-1* in a sensitive background<br>to insert *Spk-1$^{ORF1[ATG>TTG]}$* in a sensitive background<br>to insert *Spk-1$^{ORF2[ATG>TTG]}$* in a sensitive background<br>to insert *Spk-1$^{ORF2[ATG>ATGG]}$* in sensitive background<br>to insert *Spk-1$^{ORF2[mut5]}$* in sensitive background	
1577	AGCATTTTCACCTTGGCCGTGAG	C
1578	GAGTCCCGTTATTGCCGTTTGACC	C
1579	GATGGTTTGGCCTCTTGGACGAGT	L
1613	CTCACGGCCAAGGTGAAAATGCTCATGCGCTTCGCATATACAG	L
1581	GGTCAAACGGCAATAACGGGACTCTGCTATTGCCGTCTTTGTCATGTT	R
1582	TACTGCTCCAGAGTTGGGGTCGAG	R
1583	TTGGTCGTTGTTGCTTGTTGTTGG	N
1584	ACCGTGGGATTTTGCATCCTTTG	N
v235 (hph)	to delete the 5' half of *Spk-1 ORF1*	
12	AACTGATATTGAAGGAGCATTTTTTGG	C
13	AACTGGTTCCCGGTCGGCAT	C
1755	AGGTGAGTTGGAAGGAGCGGAAC	L
1756	AAAAAATGCTCCTTCAATATCAGTTTGGTGACGCAAGTGGTTTCTTTT	L
1757	GAGTAGATGCCGACCGGGAACCAGTTCTCAGTCTTGGTCGTTGCCCTGT	R
1758	CGTATTTTCTTTCGCTTGCCTCTTC	R
1759	TTCCTCAAGGCGGGCTACGTTC	N
1760	TGTTGTTTTCAGACTATCCGCTCCA	N
v236 (hph)	to delete the 3' 2/3 of *Spk-1 ORF2*	
12	AACTGATATTGAAGGAGCATTTTTTGG	C
13	AACTGGTTCCCGGTCGGCAT	C
1761	TGCCTAGCAAACGGTAAGCATCAA	L
1762	AAAAAATGCTCCTTCAATATCAGTTATGGGGTGTTCGACGGAAAAGA	L
1763	GAGTAGATGCCGACCGGGAACCAGTTGCGAAGAGGCAAGCGAAAGAAAA	R
1764	CGTTCACGAGAGAGAAACGGTCA	R
1765	ATCCTTCTCCCTCCCACCAAACA	N
1766	AGGCTTCCCTTCCACTCACTTCC	N

Nine transformation vectors were constructed for this report: v134, v178, v205a–v205e, v235, and v236. The center fragment for each vector is listed within parentheses next to each vector name (e.g. *hph*). C, center fragment primer; L, left flank primer; R, right flank primer; N, nested primer.
